# The role of group III/IV afferent feedback in the ventilatory, pressor and metabolic responses to exercise in normoxia and hypoxia

**DOI:** 10.1113/JP290914

**Published:** 2026-06-19

**Authors:** Elliott J. Jenkins, Phuong L. Ha, Connor A. Howe, Jay M. J. R. Carr, Justin A. Monteleone, Andrew J. M. Douglas, Abigail Nicholson, Andrew Steele, Brydie Lemkes, Jodie L. Koep, L. Madden Brewster, Jennifer Duffy, Mathew I. B. Debenham, Tena Monaghan, Declan Isaak, Jacob McCormick, Brian H. Dalton, Chris J. McNeil, Jared Baylis, David MacLeod, Mike Stembridge, Markus Amann, Philip N. Ainslie, Travis D. Gibbons

**Affiliations:** 1 School of Sport and Health Sciences, Cardiff Metropolitan University, Cardiff, Wales, UK; 2 School of Health and Exercise Sciences, University of British Columbia, Kelowna, British Columbia, Canada; 3 Institute of Sport Science, University of Innsbruck, Innsbruck, Austria; 4 Institute of Mountain Emergency Medicine, Eurac Research, Bolzano, Italy; 5 Centre for Heart, Lung and Vascular Health, University of British Columbia, Kelowna, British Columbia, Canada; 6 School of Physical Education, Sport and Exercise Sciences, University of Otago, Dunedin, New Zealand; 7 Interior Health Authority, Kelowna, British Columbia, Canada; 8 Department of Emergency Medicine, University of British Columbia, Vancouver, British Columbia, Canada; 9 Department of Anesthesiology, Duke University Medical Center, Durham, North Carolina, USA; 10 Department of Anesthesiology, University of Utah, Salt Lake City, Utah, USA; 11 Department of Biological Sciences, Northern Arizona University, Flagstaff, Arizona, USA

**Keywords:** exercise, group III/IV muscle afferents, hypoxia, oxygen transport

## Abstract

Group III/IV muscle afferents play a prominent role in ensuring appropriate arterial oxygenation during exercise. Exercise in hypoxia poses a major cardiorespiratory challenge due to reduced inspired oxygen pressure (P_I_O_2_). Although humans adapt powerfully to hypoxia, the contribution of group III/IV afferents to ventilatory, pressor and metabolic responses to exercise in chronic hypoxia remains unknown. Fourteen healthy participants completed cycling trials with (placebo) and without (25 μg intrathecal fentanyl) intact group III/IV afferent feedback in three settings: (1) normoxia, P_I_O_2_ = 139 mmHg; (2) acute hypoxia, P_I_O_2_ = 90 mmHg; and (3) chronic hypoxia, after 7–9 days of acclimatisation to 3800 m, P_I_O_2_ = 90 mmHg. Participants completed three 3-min stages at matched absolute intensities corresponding to 45%, 60% and 75% sea-level maximal aerobic power. Ventilation, pulmonary gas exchange, arterial blood gases and blood pressure were measured. Fentanyl suppressed exercise hyperpnoea similarly across environments (*P* < 0.001) but produced larger reductions in arterial oxygenation in hypoxia, lowering saturation by ~3%–5% and content by ~0.7–1.0 ml dL^−1^ (*condition*×*P*_*I*_*O*_*2*_, *P* ≤ 0.016). Fentanyl also blunted the exercise-induced increase in mean arterial pressure, with a greater blood pressure–lowering effect of −10 to 13 mmHg observed in hypoxia (*condition*×*P*_*I*_*O*_*2*_, *P* = 0.021). In contrast fentanyl increased circulating lactate and hydrogen ion concentration ([H^+^]) (*P* < 0.001), an effect accentuated during acute hypoxia (lactate: +1–2 mmol L^−1^, [H^+^]: +2–5 nmol L^−1^) but attenuated after acclimatisation (*interactions*, *P* ≤ 0.034). Thus group III/IV afferent feedback becomes more consequential in hypoxia, as small perturbations in ventilatory control and perfusion pressure have amplified consequences for oxygen transport.

## Graphical Abstract

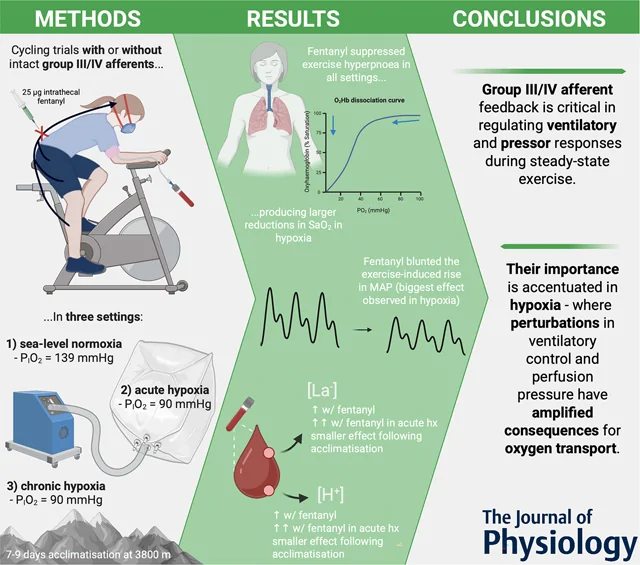

Participants completed cycling exercise with (placebo) and without (25 μg intrathecal fentanyl) intact group III/IV muscle afferent feedback in three settings: normoxia, acute hypoxia and chronic hypoxia after 7–9 days of acclimatisation (~3800 m). Blocking afferent feedback suppressed exercise hyperpnoea across all environments, but resulted in larger reductions in arterial oxygenation in hypoxia. Fentanyl also attenuated the exercise-induced increase in mean arterial pressure, with a greater blood pressure–lowering effect observed in hypoxia. In contrast circulating lactate and hydrogen ion concentration ([H^+^]) increased, particularly during acute hypoxia, with partial attenuation after acclimatisation. These findings demonstrate that group III/IV afferent feedback plays a critical role in maintaining arterial oxygenation and perfusion pressure during exercise, with heightened physiological importance in hypoxia where small disruptions to ventilatory control and cardiovascular regulation have amplified consequences for oxygen transport.

## Introduction

Neural feedback from unmyelinated and thinly myelinated group III and IV muscle afferent fibres plays an essential role in regulating the ventilatory and cardiovascular responses to physical activity (Amann et al., 2010; Thurston et al., 2023). Specifically feedback from these group III/IV muscle afferents ensures normal exercise hyperpnoea (Amann et al., 2010), pulmonary gas exchange (Iannetta et al., 2024) and – via their role in the exercise pressor reflex – suitable arterial pressure (Thurston et al., 2023). Collectively these responses ensure a matching of oxygen delivery to oxygen demand.

Exercise in hypoxia presents a considerable cardiorespiratory challenge, fundamentally caused by decreased oxygen availability when demand is high. Decreased inspired oxygen pressure (P_I_O_2_) requires exaggerated exercise hyperpnoea to defend alveolar oxygen pressure ($P_{{AO}_{2}}$), which can lead to exertional dyspnoea (Chronos et al., 1988). However even with this augmented ventilatory response, the hypoxia-induced reduction in $P_{{AO}_{2}}$ constrains the alveolar–pulmonary capillary oxygen pressure gradient, resulting in a diffusion limitation at mild-to-moderate exercise intensities (Dempsey et al., 1971). Given group III/IV afferents have a dominant role in mediating appropriate exercise hyperpnoea, pulmonary gas exchange and exertional dyspnoea (Gagnon et al., 2012; Iannetta et al., 2024), their function during exercise in hypoxia is critical for appropriate oxygen transport. The precision of ventilatory responses to exercise in hypoxia is particularly important given small decreases in arterial oxygen pressure $\left(P_{{aO}_{2}}\right)$ have exaggerated effects on oxygen saturation $\left(S_{{aO}_{2}}\right)$ due to the shape of the oxygen–haemoglobin disassociation curve and therefore total oxygen content $\left(C_{{aO}_{2}}\right)$.

There is evidence to suggest that group III/IV muscle afferents interact synergistically with carotid chemoreceptors under acute hypoxic conditions (Wan et al., 2020a; Wan et al., 2020b), potentially enhancing oxygen transport when availability is limited. Similar to carotid chemoreceptors group III/IV muscle afferents are sensitive to hypoxia, with increased discharge rates observed during hypoxaemic muscle perfusion in anaesthetised cats (Hill et al., 1992). However it is likely that this heightened discharge rate in hypoxia reflects increased intramuscular metabolic strain imposed by reduced muscle oxygen delivery, such that hypoxia itself contributes little to the activation of group III/IV muscle afferents (Amann & Kayser, 2009). Acclimatisation to chronic hypoxia results in several coordinated adaptations that act to restore $C_{{aO}_{2}}$ despite a persistent reduction in P_I_O_2_. This restoration occurs initially via rapid hyperventilation, which increases $P_{{AO}_{2}}$ and $P_{{aO}_{2}}$ (Forster et al., 1971). With more prolonged exposure, oxygen is concentrated in the blood first via contraction of plasma volume (Roche et al., 2022), and thereafter via expansion of total red cell volume (Siebenmann et al., 2015; Siebenmann et al., 2024; Stickney & Van Liere, 1953). Although the restoration of $C_{{aO}_{2}}$ does not appear to attenuate peripheral locomotor muscle fatigue during exercise in chronic hypoxia (Amann et al., 2013), these adaptations may mitigate some of the muscle metabolic strain imposed by acute hypoxia (Bejder et al., 2025; Hogan et al., 1992), thereby altering the activation and, consequently, the contribution of group III/IV afferent feedback during exercise. Limited data in rats exposed to 45 days of 9.5% oxygen suggest that chronic hypoxia desensitises type III/IV afferents to fatiguing stimuli and lactic acid infusion (Dousset et al., 2001). However these data have been appropriately criticised as they do not take into account the observation that baseline group III/IV afferent discharge is already considerably elevated in hypoxia and the net discharge in response to activating stimuli is still larger in chronic hypoxia compared to rats in normoxia (Amann & Kayser, 2009; Hill et al., 1992).

In a randomised, placebo-controlled design, we examined whether acute and chronic hypoxic exposures alter the functional contribution of group III/IV muscle afferents to ventilatory, pressor and metabolic responses during steady-state exercise, using intrathecal fentanyl to attenuate afferent feedback. Exercise was performed at matched absolute workloads within three experimental settings: (1) sea-level normoxia, (2) sea-level acute hypoxia and (3) high-altitude chronic hypoxia after 7–9 days of acclimatisation. Given that exercise in hypoxia occurs on the steep portion of the oxygen–haemoglobin dissociation curve, we hypothesised that intrathecal fentanyl–induced suppression of exercise hyperpnoea would have disproportionately greater consequences for $S_{{aO}_{2}}$ and $C_{{aO}_{2}}$. We further hypothesised that group III/IV afferent feedback would make a greater contribution to the maintenance of arterial pressure during exercise in hypoxia. Finally due to the heightened metabolic strain imposed by acute hypoxia, we hypothesised that suppression of group III/IV muscle afferent feedback would lead to greater increases in circulating lactate and hydrogen ion concentration in this setting than in normoxia and that this effect would be attenuated after acclimatisation, secondary to adaptations in $C_{{aO}_{2}}$.

## Methods

### Ethical approval

Ethical approval was granted by the University of British Columbia Research Ethics Board (H22–01091) and conformed to the *Declaration of Helsinki* (2013), with prior registration at ClinicalTrials.gov (ID: NCT05619887). Prior to participation, written informed consent was obtained from all participants.

### Participants

Seventeen healthy participants (7 females) volunteered to take part in the study (age, 27.9 ± 5.3 years; body mass, 69.8 ± 9.6 kg; height, 173.5 ± 9.9 cm; maximal oxygen consumption $\left({\dot{V}}_{O_{2}max}\right)$, 50.8 ± 7.4 mL·min^−1^·kg^−1^). Participants were non-smokers, without cardiovascular or respiratory disease, and each refrained from vigorous exercise and alcohol consumption for 24 h before participation. The investigation consisted of sea-level and high-altitude visits; three participants (1 female) were unavailable for sea-level testing, whereas the remaining 14 participated in both environments.

### Experimental design

The study utilised a repeated-measures doubled-blind cross-over design in which participants completed two identical cycling tests at sea level (Kelowna, BC, Canada: 340 m) and repeated them 3 months later, after 7–9 days of acclimatisation at high altitude (Barcroft Station, White Mountain, CA, USA: 3800 m). This timeframe was selected to coincide with substantial restoration of $C_{{aO}_{2}}$, consistent with evidence that the major haematological adaptations to altitude occur within ~2 weeks at comparable altitude (Lundby et al., 2004). Within each experimental visit participants were administered subcutaneous saline at L3–L5 (CON) or fentanyl intrathecally at L3–L5 (FENT) to isolate the role of locomotor group III/IV muscle afferents on exercise responses in hypoxia. Cycling conditions were separated by ≥3 days to limit potential carry-over effects, whereas time of day was matched within each participant (±1 h) to minimise diurnal effects.

### Experimental protocol

During a preliminary lab visit participants completed a maximal incremental exercise test on a cycle ergometer (Kickr, Wahoo, Atlanta, GA, USA) to determine ${\dot{V}}_{O_{2}\text{max}}$ and establish constant load exercise intensities. ${\dot{V}}_{O_{2}\text{max}}$ tests were performed once each at sea level and high altitude, with the testing conducted at sea level used to determine the constant load work rates corresponding to 45%, 60% and 75% of normoxic ${\dot{V}}_{O_{2}\text{max}}$. G ${\dot{V}}_{O_{2}\text{max}}$ was reduced by ~23% at high altitude, mean power outputs of 90, 135 and 185 W at sea level corresponded to ~60%, 75% and 90% of high-altitude ${\dot{V}}_{O_{2}\text{max}}$.

**Figure 1.**
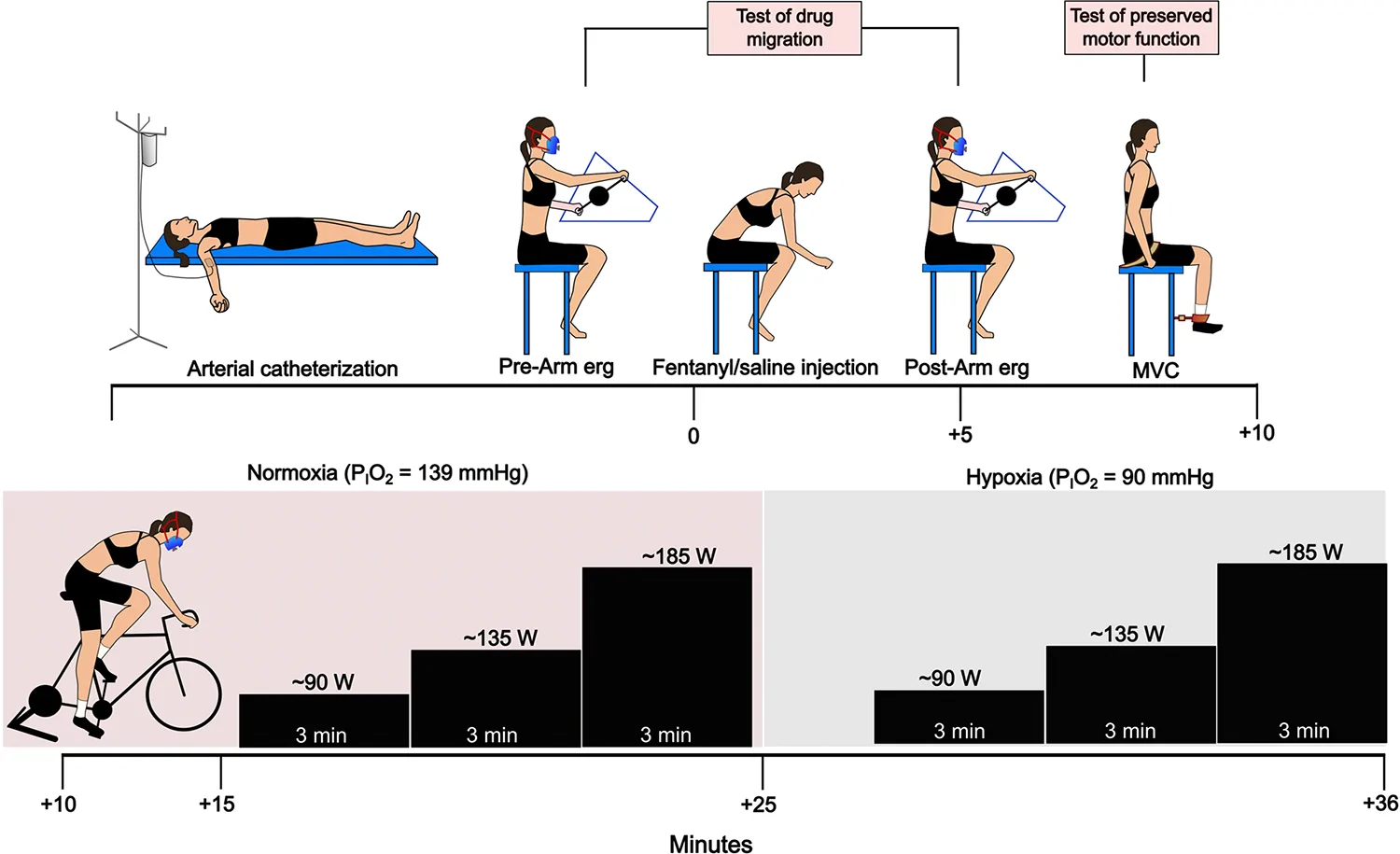
Protocol schematic Participants completed trials at sea level (Kelowna, Canada, 340 m) and after 7–9 days at high altitude (White Mountain, CA, USA, 3800 m). After arterial catheterisation, neuromuscular testing (including maximal voluntary contraction (MVC) of the quadriceps) and arm ergometry, saline (CON) or fentanyl (FENT) was administered intrathecally at L3–L5. Cycling consisted of three 3-min bouts at mean external power outputs of 90, 135 and 185 W (~45%, 60% and 75% normoxic ${\dot{V}}_{O_{2}max}$ at sea level). The sequence was performed first in normoxia ($F_{{IO}_{2}}=0.209$, P_I_O_2_ ≈ 139 mmHg, $S_{{aO}_{2}}\sim 97\%$) and immediately repeated in hypoxia ($F_{{IO}_{2}}=0.135$, P_I_O_2_ ≈ 90 mmHg, $S_{{aO}_{2}}\;\sim 89\%$), where the same work rates equated to ~60%, 75% and 90% of hypoxic ${\dot{V}}_{O_{2}max}$. At high altitude the order of exposure was preserved: participants cycled first in hyperoxia (sea-level equivalent; $F_{O_{2}}=0.305$, P_I_O_2_ ≈ 139 mmHg) and then in ambient hypoxia ($F_{O_{2}}=0.209$, P_I_O_2_ ≈ 90 mmHg). Blood samples and perceptual ratings were obtained at the end of each stage, with cardiorespiratory variables recorded continuously.

On experimental trial days, following brachial artery cannulation, participants rested in a seated position for 8 min (resting baseline) and then completed 3 min of arm ergometry before and 2–3 min after intrathecal administration of fentanyl (FENT) or subcutaneous administration of normal saline (CON). A neuromuscular assessment was then conducted before participants were seated on a cycle ergometer. The exercise protocol began with 3 min of seated rest while breathing room air, followed by 3-min cycling bouts at 45%, 60% and 75% sea-level ${\dot{V}}_{O_{2}\text{max}}$ (mean external power: 90, 135, 185 W). Immediately upon completing the final bout, participants were switched to a Douglas bag delivering a hypoxic gas mixture ($F_{{IO}_{2}}=0.135$, P_I_O_2_ = 90 mmHg, equivalent to the P_I_O_2_ expected at 3800 m). After a further 3 min of seated rest, participants repeated the 3-min exercise bouts at the same external work rates, that is, 90, 135 and 185 W, corresponding to a higher proportion of hypoxic ${\dot{V}}_{O_{2}\text{max}}$. At high altitude the order of hypoxic exposure was preserved, meaning participants breathed first from a Douglas bag containing hyperoxic gas ($F_{{IO}_{2}}=0.305$, P_I_O_2_ = 139 mmHg) to mimic sea-level inspired oxygen, before switching to room air for the hypoxic bouts. It is important to note that seated ‘rest’ values were collected immediately before exercise began but after several preceding procedures (e.g. arm ergometry, neuromuscular assessments, intrathecal injection). As such, they likely reflect a mildly elevated physiological state and are best interpreted as ‘pre-exercise values’ rather than true resting values. This particularly applies to sea-level hypoxia and high-altitude hypoxic stages which follow the normoxic/hyperoxic exercise bouts. Blood samples and perceptual responses were obtained at the end of each exercise stage, while cardiorespiratory responses were recorded continuously (see Fig. 1).

### Measurements

#### Blood sampling.

A local anaesthetic (1% lidocaine) was injected superficial to the brachial artery prior to cannulation. Using ultrasound guidance, the brachial artery was cannulated with a 20-gauge cannula (Arrow, Markham, ON, Canada), with the alternate arm cannulated during the subsequent condition. The cannula was connected in series to a wasteless sampling system (VAMP, Edwards Lifesciences, Irvine, CA, USA) and a pressure transducer, which was levelled to the height of the right atrium for continuous beat-by-beat intra-arterial blood pressure monitoring (ADInstruments, Dunedin, NZ) providing measurements of systolic blood pressure (SBP), diastolic blood pressure (DBP) and mean arterial pressure (MAP). Blood samples were analysed immediately for pH, $P_{{aO}_{2}},\;P_{{aCO}_{2}},\;S_{{aO}_{2}}$, bicarbonate concentration ([HCO_3_^−^]), hydrogen ion concentration ([H^+^]), haematocrit (Hct), haemoglobin concentration ([Hb]) and blood glucose and lactate concentrations using an ABL90 FLEX blood gas analyser (Radiometer, Copenhagen, Denmark). Rectal temperature was measured continuously using a T-type general-purpose sterile thermistor (ADInstruments) inserted ~15 cm beyond the sphincter. Arterial blood gas measurements were temperature corrected to the rectal temperature at the time of sampling using previously derived constants and logarithmic equations (Severinghaus, 1966).

#### Exercise responses.

Ventilation $\left({\dot{V}}_{E}\right)$ and pulmonary gas exchange were measured breath by breath at rest and throughout exercise stages via a metabolic cart (Vyntus CPX, Vyaire Medical, Mettawa, IL, USA). Heart rate (HR) was measured continuously (beat to beat) from the R–R period of ventricular depolarisation (H10, Polar, Kempele, Finland) and converted to digital signal for storage on computer software (SentrySuite, Vyaire Medical). Additionally in the final 15 s of each steady-state exercise stage, participants were asked to give subjective scores of dyspnoea and leg discomfort using Borg’s modified CR10 scale (Borg, 1998).

#### Intrathecal fentanyl.

Participants were seated in a flexed position, and 0.025 mg of fentanyl was administered intrathecally at the L3–L5 vertebral interspace, as has been detailed previously (Amann et al., 2009). Fentanyl remains active for ~60 min after administration and blocks ~50% of the feedback from the legs without impairing muscle strength (Amann et al., 2010; Hureau et al., 2019). To determine whether fentanyl migrated beyond the cervical spinal cord and influenced medullary *μ*-opioid receptors involved in breathing, we assessed ventilatory responses to a standardised arm-ergometry task (30 W for 3 min; Monark 881E, Monark Sports & Medical, Vansbro, Sweden) before and after drug administration. This evaluation was necessary, as cephalad migration of fentanyl into brain regions controlling respiration could complicate data interpretation (Lalley, 2008). Ventilatory responses to arm cycling before and after fentanyl administration were assessed on an individual basis. Reductions greater than 2 SD from the breath-by-breath recording of minute ventilation during the final minute of arm cycling observed in CON were considered evidence of a cephalic migration of fentanyl (Thurston et al., 2023). HR and arterial blood pressure were also monitored during the arm-ergometry task; however due to the positioning of the arterial catheter and movement artefact associated with upper-body exercise, blood pressure signals were not considered sufficiently reliable for analysis. To ensure protocol consistency across testing days, the same arm-ergometry assessment was performed following saline administration.

### Statistical analyses

Analyses were conducted in R (version 4.5.2, R Foundation for Statistical Computing, Vienna, Austria) using linear mixed-effects models, implemented using the lme4, lmerTest and emmeans packages. Condition (FENT, CON), P_I_O_2_ (normoxia, hypoxia), acclimatisation (sea level, high altitude) and intensity (~90, ~135, ~185 W) were included as fixed effects, with participant modelled as a random factor. Rest data were not included in the statistical models and appear only in figures for contextual reference. Paired (within-subject) *t* tests were performed on seated rest data to confirm the absence of physiological effects of intrathecal fentanyl under resting conditions. *A priori* planned contrasts tested the fentanyl–control delta for (1) acute hypoxia (sea-level hypoxia *vs*. sea-level normoxia, *P*_*I*_*O*_*2*_ effect) and (2) acclimatisation (high-altitude hypoxia *vs*. sea-level hypoxia, *acclimatisation* effect). Because neither *condition* × *P*_*I*_*O*_*2*_ × *intensity* nor *condition* × *acclimatisation* × *intensity* interactions were present, fentanyl–control effects are averaged across intensities. Main effects of intensity are reported separately. *P*-values were calculated using the Satterthwaite method with *α* = 0.05. Results are presented as observed mean differences (Δ; fentanyl–control) with 95% confidence intervals (CIs) and units; data are mean ± SD unless stated otherwise. Figures and data were obtained using GraphPad Prism (version 10.6.1, GraphPad Software Inc., San Diego, CA, USA).

## Results

### Tests of cephalad drug migration

Each participant’s ventilatory response to arm cycling in FENT was within 2 SD of CON. The mean minute ventilation during arm cycling at 30 W (~41 ± 8 L·min^−1^) was not different between conditions (*P* = 0.111). HR during arm cycling (~123 ± 1.3 bpm) was also not different between CON and FENT (*P* = 0.148). These findings argue against a direct effect of fentanyl on cortical opioid receptors and are consistent with the absence of cephalad drug migration. For maximal voluntary contraction of the knee extensors, there was no difference between CON and FENT at sea level (*P* = 0.207) or high altitude (*P* = 0.509) prior to cycling, indicating that efferent nerve activity and quadriceps strength were unaffected by intrathecal fentanyl.

Additionally intrathecal fentanyl had no effect on any measured cardiorespiratory, metabolic or perceptual variables during seated resting conditions (i.e. prior to the onset of cycling exercise) at either sea level or high altitude. Unless otherwise stated all subsequent results refer to steady-state exercise responses.

### Inspired oxygen partial pressure control

P_I_O_2_ was reduced at sea level to simulate hypoxia equivalent to 3800 m altitude, allowing distinction between the effects of acute and chronic hypoxic exposure. Room air P_I_O_2_ averaged 139 ± 1 mmHg at sea level and 91 ± 1 mmHg at high altitude, and was closely matched during gas administration (sea-level hypoxia: 90 ± 1 mmHg). Importantly P_I_O_2_ did not differ between control and fentanyl conditions (*P* = 0.461; Table 1) at each altitude.

### Ventilatory control during steady-state exercise

#### Exercise hyperpnoea.

**Figure 2.**
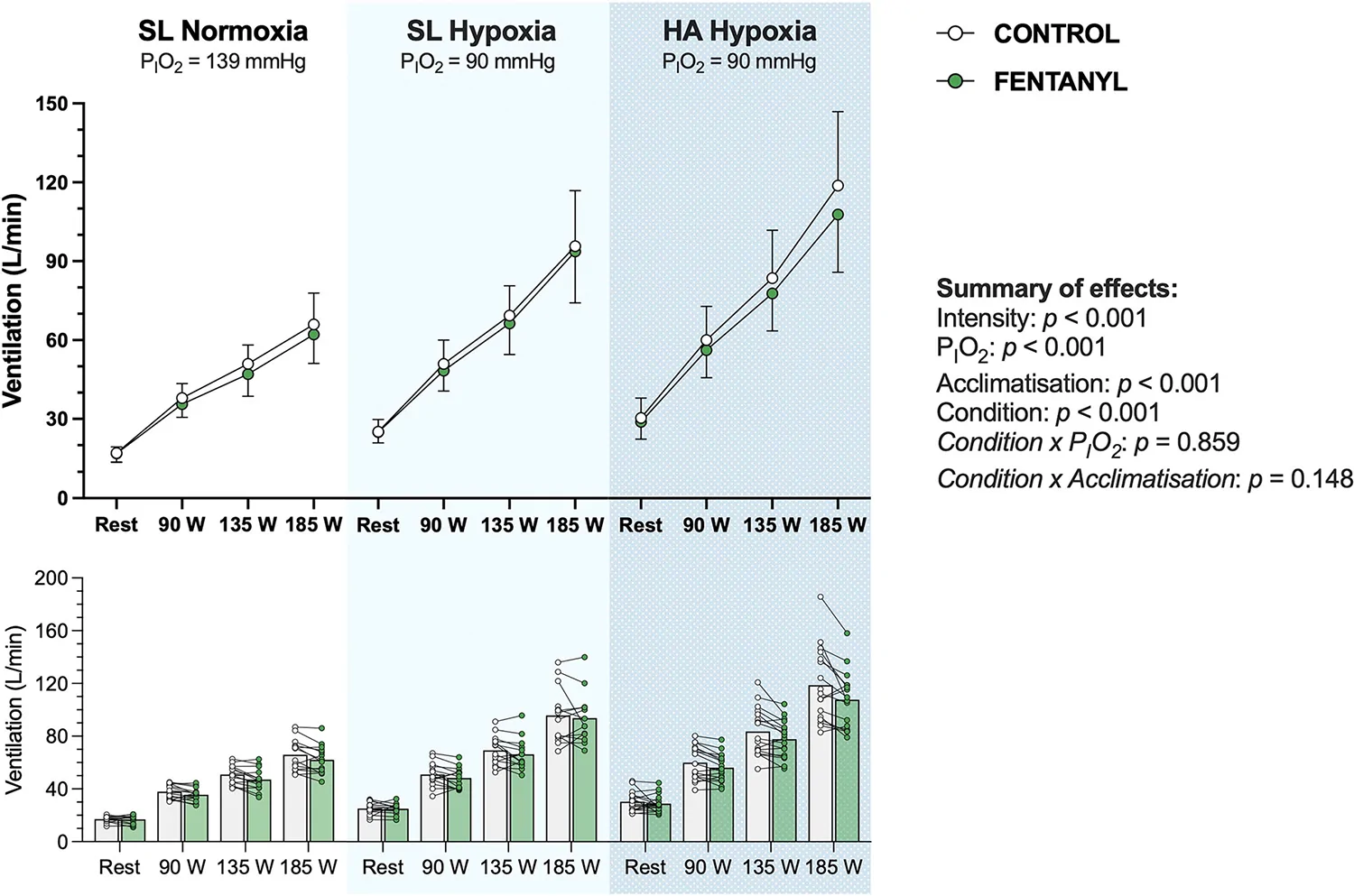
Ventilation (${\dot{V}}_{E}$) at rest and during steady-state exercise Top panels show group mean ± SD responses across exercise intensity (rest, 90, 135 and 185 W), condition (control, fentanyl), inspired oxygen partial pressure (normoxia: 139 mmHg, hypoxia: 90 mmHg) and acclimatisation status (SL, sea level; HA, high altitude). Bottom panels show individual paired data points between control and fentanyl conditions, with bars representing group means at each intensity.

When participants breathed room air at sea level, ${\dot{V}}_{E}$ increased with exercise intensity (*P* < 0.001; Table 1; Fig. 2). Acute hypoxia further increased exercise hyperpnoea by an average of 18 L·min^−1^ (*P* < 0.001; [15, 20]), and this was further increased by 11 L·min^−1^ after acclimatisation (*P* < 0.001; [9, 14]). Increases in ${\dot{V}}_{E}$ during acute hypoxia and after acclimatisation were mediated almost exclusively by increases in breathing frequency (Fb; Table 1). Intrathecal fentanyl suppressed exercise hyperpnoea in all environments by 3 L·min^−1^ (*P* < 0.001; [−5, −2]), with no differential effects in acute hypoxia or after acclimatisation. Interestingly the effect of intrathecal fentanyl on suppressing Fb was greater after acclimatisation to high altitude (*condition* × *acclimatisation*, *P* = 0.008; Table 1).

#### Exercise hyperventilation.

**Figure 3.**
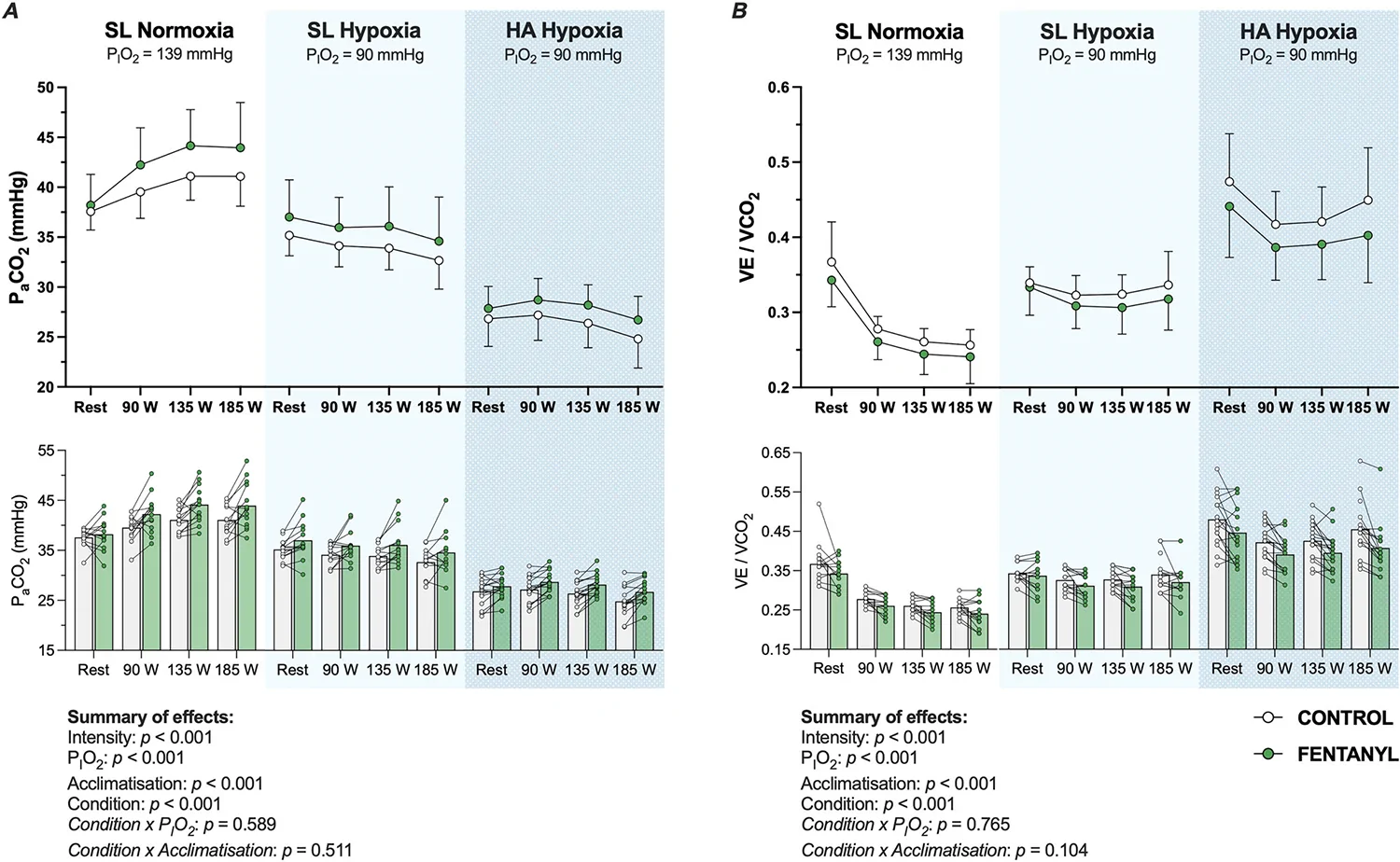
*A*, arterial CO_2_ tension ($P_{{aCO}_{2}}$) and *B*, the ventilatory equivalent for CO_2_ (${\dot{V}}_{E}/{\dot{V}}_{{CO}_{2}}$) at rest and during steady-state exercise Top panels show group mean ± SD responses across exercise intensity (rest, 90, 135 and 185 W), condition (control, fentanyl), inspired oxygen partial pressure (normoxia: 139 mmHg, hypoxia: 90 mmHg) and acclimatisation status (SL, sea level; HA, high altitude). Bottom panels show individual paired data points between control and fentanyl conditions, with bars representing group means at each intensity.

During room air breathing at sea level, $P_{{aCO}_{2}}$ increased with exercise intensity (*P* < 0.001; Table 1; Fig. 3A). On the contrary, exercise in acute hypoxia caused $P_{{aCO}_{2}}$ to progressively decrease (Δ = −6 mmHg *vs*. normoxia, *P* <0.001; [−6.6, −5.4]), and this was even greater after acclimatisation (Δ = −8 mmHg *vs*. acute hypoxia, *P* < 0.001; [−8.8, −7.6]). Across all environments intrathecal fentanyl increased $P_{{aCO}_{2}}$ by 1.9 mmHg (*P* < 0.001; [1.5, 2.3]). Intrathecal fentanyl did not differentially affect $P_{{aCO}_{2}}$ in acute hypoxia or after acclimatisation (*interactions, P* ≥ 0.511). As expected, the ventilatory equivalent for CO_2_ $\left({\dot{V}}_{E}/{\dot{V}}_{{CO}_{2}}\right)$ followed a similar pattern (Fig. 3B).

### Pulmonary gas exchange and arterial oxygenation

#### Alveolar and arterial oxygen tension.

When participants breathed room air at sea level, $P_{{AO}_{2}}$ increased with exercise intensity (*P* < 0.001; Table 1). Acute hypoxia substantially reduced $P_{{AO}_{2}}$ (Δ = −34 mmHg *vs*. normoxia, *P* < 0.001; [−35, −33]). The decrease in $P_{{AO}_{2}}$ was partially restored after acclimatization (Δ = 7 mmHg *vs*. acute hypoxia, *P* < 0.001; [5.9, 8.1]). Across all environments intrathecal fentanyl reduced $P_{{AO}_{2}}$ by 1.6 mmHg (*P* < 0.001; [−2.3, −0.8]), with no differential effects in acute hypoxia or after acclimatisation (*interactions*, *P* ≥ 0.41).

When participants breathed room air at sea level, $P_{{aO}_{2}}$ decreased with exercise intensity (*P* < 0.001; Table 1). As expected, acute hypoxia substantially reduced $P_{{aO}_{2}}$ (Δ = −39 mmHg *vs*. normoxia, *P* < 0.001; [−41, −38]). The decrease in $P_{{aO}_{2}}$ was partially restored after acclimatisation (Δ = 7.9 mmHg *vs*. acute hypoxia, *P* < 0.001; [6.6, 9.2]). Across all environments intrathecal fentanyl reduced $P_{{aO}_{2}}$ by 2.5 mmHg (*P* < 0.001; [−3.4, −1.6]), with no differential effects in acute hypoxia or after acclimatisation (*interactions*, *P* ≥ 0.144).

#### Alveolar–arterial oxygen difference.

**Figure 4.**
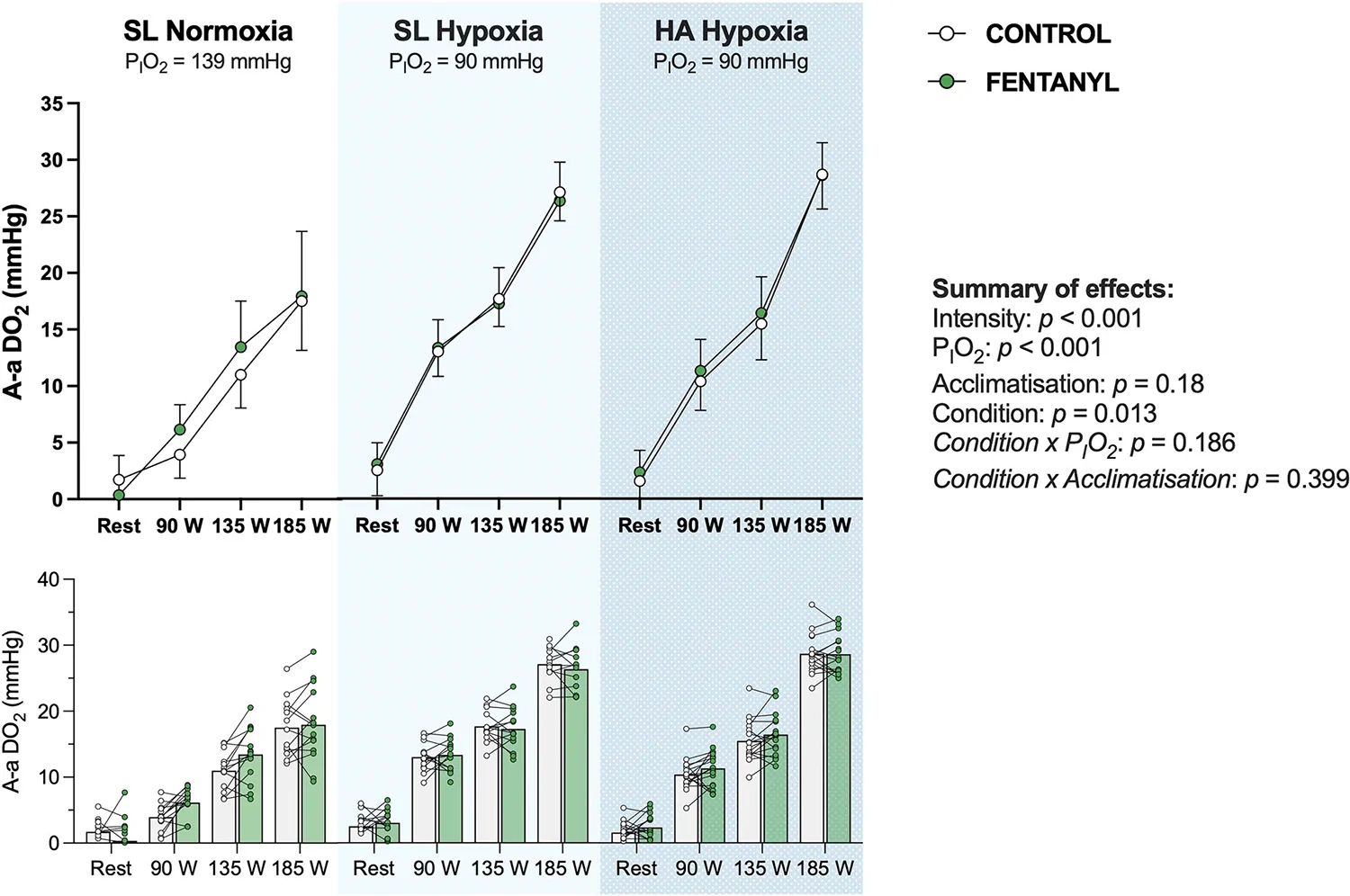
Alveolar–arterial oxygen difference (A–a DO_2_) during rest and steady-state exercise Top panels show group mean ± SD responses across exercise intensity (rest, 90, 135 and 185 W), condition (control, fentanyl), inspired oxygen partial pressure (normoxia: 139 mmHg, hypoxia: 90 mmHg) and acclimatisation status (SL, sea level; HA, high altitude). Bottom panels show individual paired data points between control and fentanyl conditions, with bars representing group means at each intensity.

When participants breathed room air at sea level, higher exercise intensities increased the alveolar–arterial oxygen difference (A–aDO_2_; *P* < 0.001; Table 1; Fig. 4). The exercise-induced increase in A–aDO_2_ was 5.9 mmHg greater in acute hypoxia (*P* < 0.001; [5.0, 6.8]) and remained similarly elevated with acclimatisation (*P* = 0.18 *vs*. acute hypoxia). Across all environments intrathecal fentanyl increased A–aDO_2_ by 0.8 mmHg (*P* = 0.013; [0.2, 1.4]), with effects observed primarily at low exercise intensities. The effect of intrathecal fentanyl on A–aDO_2_ was not different in acute hypoxia or after acclimatisation (*interactions*, *P* ≥ 0.186).

#### Arterial oxygenation and concentration.

**Figure 5.**
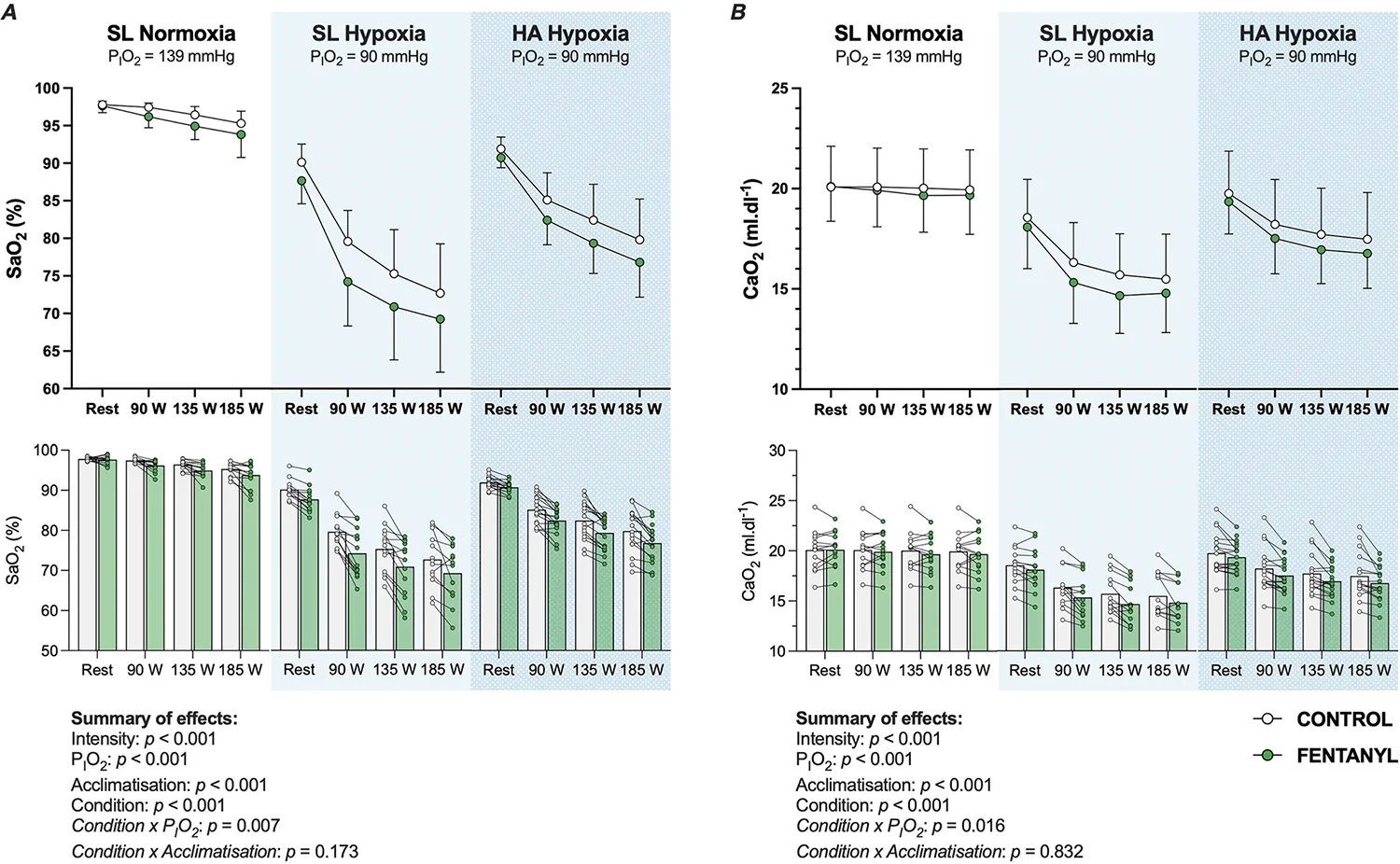
*A*, arterial oxygen saturation ($S_{{aO}_{2}}$) and *B*, arterial oxygen content ($C_{{aO}_{2}}$) during rest and steady-state exercise Top panels show group mean ± SD responses across exercise intensity (rest, 90, 135 and 185 W), condition (control, fentanyl), inspired oxygen partial pressure (normoxia: 139 mmHg, hypoxia: 90 mmHg) and acclimatisation status (SL, sea level; HA, high altitude). Bottom panels show individual paired data points between control and fentanyl conditions, with bars representing group means at each intensity.

When participants breathed room air at sea level, $S_{{aO}_{2}}$ decreased subtly with exercise intensity (*P* < 0.001; Table 1; Fig. 5A). Acute hypoxia reduced $S_{{aO}_{2}}$ by 19% (*P* < 0.001; [−20, −18]), and this desaturation was mitigated in part (+6%) after acclimatisation (*P* < 0.001 *vs*. acute hypoxia; [5, 7]). Across all settings intrathecal fentanyl reduced $S_{{aO}_{2}}$ by 2% (*P* < 0.001; [−3, −1]), and this desaturating effect of FENT was significantly greater in acute hypoxia compared to normoxia (*condition* × *P*_*I*_*O*_*2*_, *P* = 0.007). This exaggerated fentanyl-mediated desaturation persisted after acclimatisation and was not different from acute hypoxia (*condition* × *acclimatisation, P* = 0.173; Δ = −3% *vs*. CON, [−4, −1]).

The $C_{{aO}_{2}}$ response followed a similar pattern. When participants breathed room air at sea level, $C_{{aO}_{2}}$ decreased subtly with exercise intensity (*P* < 0.001; Table 1; Fig. 5B). Acute hypoxia reduced $C_{{aO}_{2}}$ by 3.8 mL dL^−1^ (*P* < 0.001; [−4.1, −3.6]), which was partly resolved (+2.1 mL dL^−1^) after acclimatisation (*P* < 0.001 *vs*. acute hypoxia; [1.9, 2.4]). Across all settings intrathecal fentanyl reduced $C_{{aO}_{2}}$ by 0.5 mL dL^−1^ (*P* < 0.001; [−0.7, −0.3]), and this effect was significantly greater in acute hypoxia compared to normoxia (*condition* × *P*_*I*_*O*_*2*_, *P* = 0.016). The effect of FENT on reducing $C_{{aO}_{2}}$ persisted after acclimatisation and was not different from acute hypoxia (*condition* × *acclimatisation, P* = 0.832).

### Cardiovascular pressor responses

**Figure 6.**
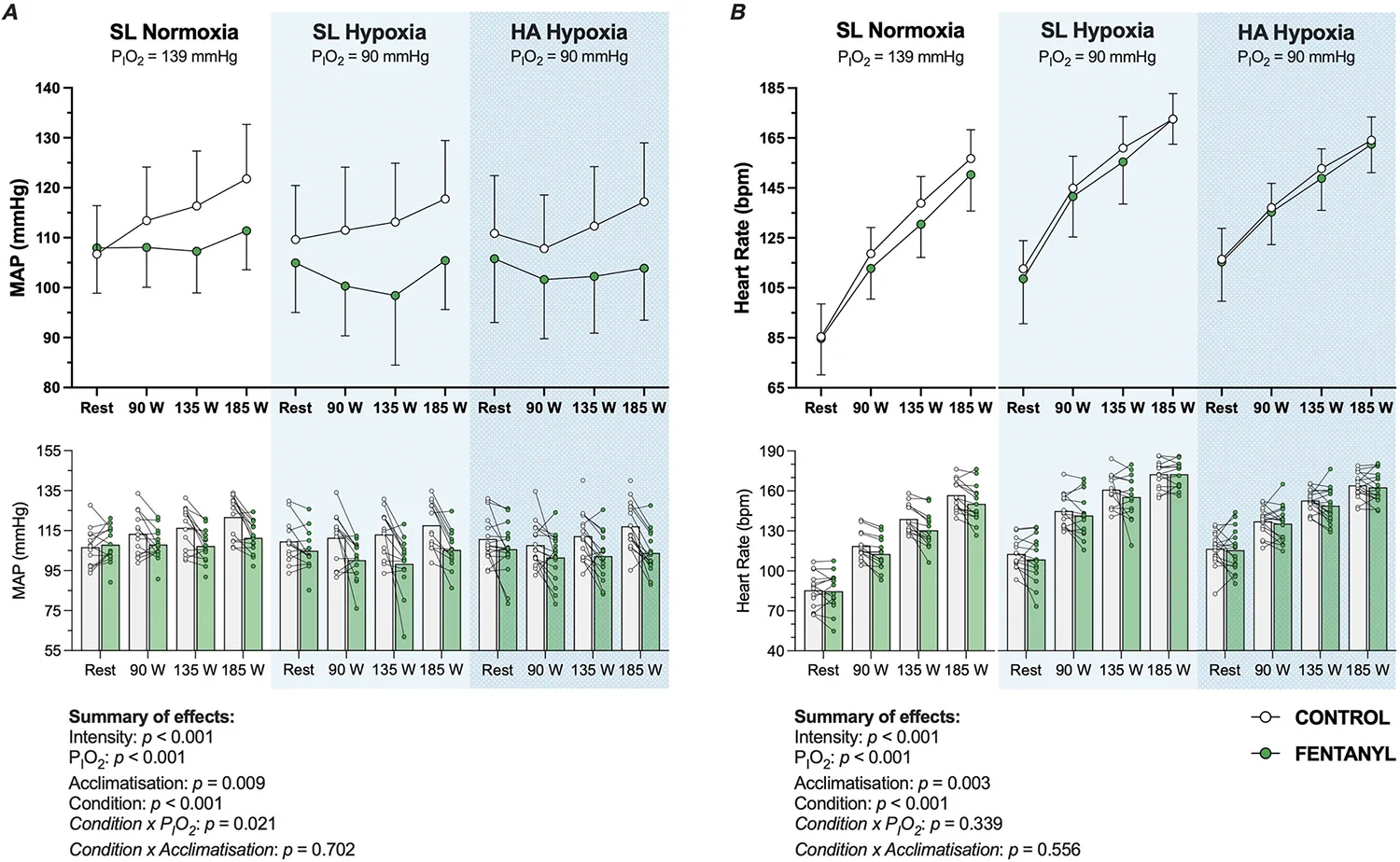
*A*, mean arterial blood pressure (MAP) and *B*, heart rate (HR) during rest and steady-state exercise Top panels show group mean ± SD responses across exercise intensity (rest, 90, 135 and 185 W), condition (control, fentanyl), inspired oxygen partial pressure (normoxia: 139 mmHg, hypoxia: 90 mmHg) and acclimatisation status (SL, sea level; HA, high altitude). Bottom panels show individual paired data points between control and fentanyl conditions, with bars representing group means at each intensity.

When participants breathed room air at sea level, MAP increased with exercise intensity (*P* < 0.001; Table 2; Fig. 6A). Acute hypoxia lowered MAP by 4.0 mmHg (*P* < 0.001; [−6.0, −2.0]), due exclusively to reductions in DBP (Table 2). Following acclimatisation, exercising MAP was subtly elevated relative to acute hypoxia (Δ = 2.6 mmHg, *P* = 0.009; [0.7, 4.5]), due to elevated DBP (Table 2). Across all environments intrathecal fentanyl had a potent blood pressure–lowering effect (Δ = −7.8 mmHg, *P* < 0.001; [−9.1, −6.5]), mediated by effects on both SBP and DBP (both *P* < 0.001). The reduction in blood pressure in FENT was significantly greater in acute hypoxia (Δ = −9.3 mmHg) than in normoxia (Δ = −4.7 mmHg; *condition* × *P*_*I*_*O*_*2*_, *P* = 0.021). After acclimatisation intrathecal fentanyl continued to lower MAP (Δ = −8.6 mmHg), driven predominantly by DBP, as the SBP response was attenuated (*condition* × *acclimatisation for SBP*: *P* < 0.001).

When participants breathed room air at sea level, HR increased with exercise intensity (*P* < 0.001; Table 2; Fig. 6B). Acute hypoxia elevated HR by 24 bpm (*P* < 0.001; [22, 26]), and this response was partially reduced after acclimatisation (Δ = −3 bpm *vs*. acute hypoxia, *P* = 0.003; [−5, −1]). Across all environments intrathecal fentanyl lowered HR by 4 bpm (*P* < 0.001; [−5, −2]). The effect of intrathecal fentanyl on HR was not different in acute hypoxia (*condition* × *P*_*I*_*O*_*2*_, *P* = 0.339) or after altitude acclimatisation (*condition* × *acclimatisation*, *P* = 0.556).

### Metabolic responses to steady-state exercise

**Figure 7.**
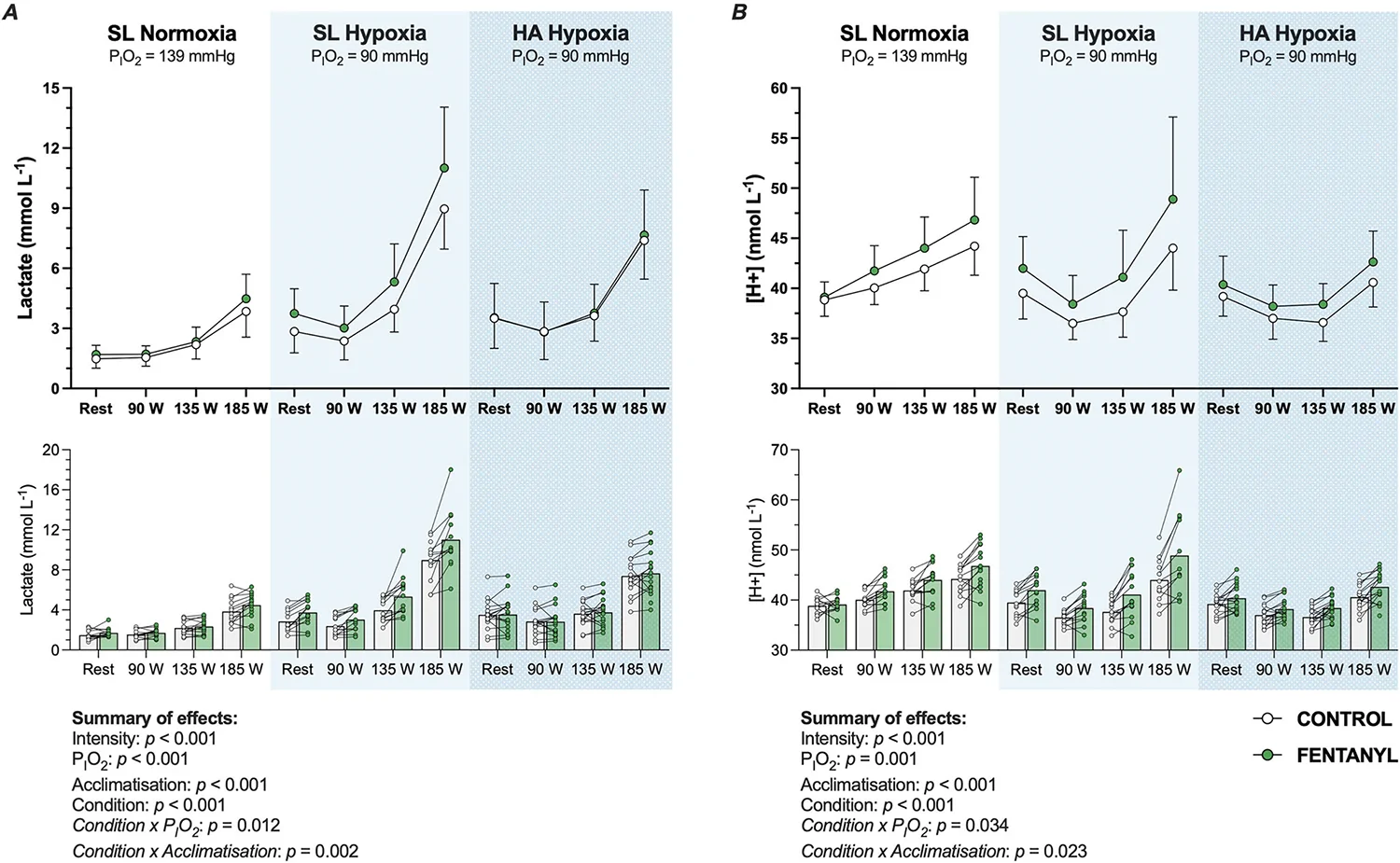
*A*, blood lactate concentration and *B*, hydrogen ion concentration [H^+^] during rest and steady-state exercise Top panels show group mean ± SD responses across exercise intensity (rest, 90, 135 and 185 W), condition (control, fentanyl), inspired oxygen partial pressure (normoxia: 139 mmHg, hypoxia: 90 mmHg) and acclimatisation status (SL, sea level; HA, high altitude). Bottom panels show individual paired data points between control and fentanyl conditions, with bars representing group means at each intensity.

When participants breathed room air at sea level, blood lactate concentration increased with exercise intensity (*P* < 0.001; Table 2; Fig. 7A), with the exercise-induced increase being 2.7 mmol L^−1^ greater in acute hypoxia (*P* < 0.001; [2.4, 3.0]). Following altitude acclimatisation, lactate during exercise was lower relative to acute hypoxia at sea level (Δ = −0.7 mmol·L^−1^, *P* < 0.001; [−1.1, −0.4]). FENT increased exercising lactate during all settings (Δ = 0.4 mmol·L^−1^, *P* < 0.001; [0.2, 0.6]). At sea level the effect of intrathecal fentanyl in increasing circulating lactate was greater in acute hypoxia (*condition* × *P*_*I*_*O*_*2*_, *P* = 0.012; Δ = 1.2 mmol·L^−1^ *vs*. CON, [0.7, 1.7]). This heightened effect of fentanyl in hypoxia was eliminated after acclimatisation (Δ = 0.2 mmol·L^−1^, *condition* × *acclimatisation, P* = 0.436; [−0.3, 0.6]).

When participants breathed room air at sea level, [H^+^] increased with exercise intensity (*P* < 0.001; Table 3; Fig. 7B); this exercise-induced acidosis was blunted in acute hypoxia (Δ = −1.1 nmol·L^−1^, *P* = 0.001; [−1.8, −0.4]). Acclimatisation to high altitude further blunted the increase in [H^+^] during exercise (Δ = 2.1 nmol·L^−1^ *vs*. acute hypoxia, *P* < 0.001; [−2.8, −1.5]). Fentanyl increased exercising [H^+^] in all settings (Δ = 2.0 nmol·L^−1^ *vs*. CON, *P* < 0.001; [1.6, 2.5]). At sea level the effect of fentanyl in increasing [H^+^] was exaggerated in acute hypoxia (*condition* × *P*_*I*_*O*_*2*_, P = 0.034; Δ = 3.2 nmol·L^−1^ *vs*. CON, [2.3, 4.1]); this effect was reversed after acclimatisation; that is the effect of fentanyl on [H^+^] was significantly attenuated relative to acute hypoxia at sea level (*condition* × *acclimatisation, P* = 0.023; Δ = 1.7 nmol·L^−1^ *vs*. CON, [0.9, 2.6]).

### Perceptual responses

When participants breathed room air at sea level, dyspnoea increased with exercise intensity (*P* < 0.001; Table 4). Acute hypoxia elevated dyspnoea by 66% (*P* < 0.001; [54, 77]), and this response was partially reduced after acclimatisation (Δ = −8%, *P* = 0.022; [−15, −1]). Across all environments intrathecal fentanyl lowered dyspnoea by 19% (*P* < 0.001; [−25, −12]). The effect of FENT on dyspnoea was not different in acute hypoxia (*condition* × *P*_*I*_*O*_*2*_, *P* = 0.496) or after altitude acclimatisation (*condition* × *acclimatisation*, *P* = 0.299).

When participants breathed room air at sea level, ratings of leg discomfort also increased with exercise intensity (*P* < 0.001). Acute hypoxia increased leg discomfort by 69% (*P* < 0.001; [50, 89]), with no further change after acclimatisation (Δ =−7%, *P* = 0.222; [−18, 4]). Intrathecal fentanyl administration reduced leg discomfort by 34% across all conditions (*P* < 0.001; [−43, −24]); the effect of intrathecal fentanyl was not different in acute hypoxia (*condition* × *P*_*I*_*O*_*2*_, *P* = 0.963) or after acclimatisation (*condition* × *acclimatisation*, *P* = 0.982).

## Discussion

This study examined how acute and chronic hypoxic exposures modify the functional contribution of group III/IV muscle afferent feedback to ventilatory, pressor and metabolic responses during steady-state exercise. The principal finding was that intrathecal fentanyl suppressed exercise hyperpnoea to a similar extent across environments, but this suppression resulted in larger reductions in $S_{{aO}_{2}}$ and $C_{{aO}_{2}}$ in hypoxia, consistent with operation on the steep portion of the oxyhaemoglobin dissociation curve. In parallel, group III/IV afferent feedback made a substantial contribution to the maintenance of arterial pressure during exercise, with a greater reliance on this reflex control in hypoxia. In contrast, the influence of group III/IV afferents on circulating lactate and [H^+^] was augmented specifically during acute hypoxia and attenuated following acclimatisation, consistent with a greater afferent contribution when muscle oxygen delivery is most constrained and a reduced contribution as $C_{{aO}_{2}}$ is partially restored. Collectively these findings indicate that the functional importance of group III/IV muscle afferent feedback is heightened in hypoxia, not via disproportionate augmentation of ventilatory drive but through the amplified consequences of small perturbations in ventilatory control and perfusion pressure for oxygen transport when oxygen availability is limited.

### Group III/IV afferents assume greater importance for arterial oxygenation during exercise in hypoxia

The equal suppression of exercise hyperpnoea by intrathecal fentanyl in normoxia, acute hypoxia and chronic hypoxia suggests that group III/IV afferents do not drive the pronounced hyperventilation observed in hypoxia. Our data therefore support the postulate that the carotid body is primarily responsible for both the acute hypoxic ventilatory response and the respiratory acclimatisation to hypoxia (Getu et al. 2025; López-Barneo et al., 2016; López-Barneo et al., 2008; Vízek et al., 1987). However it should be noted that despite equal decreases in ${\dot{V}}_{E}$ and $P_{{aO}_{2}}$ in the fentanyl condition across environments, the decrease in $S_{{aO}_{2}}$ was exaggerated in hypoxia due to positioning on the steep portion of the oxyhaemoglobin dissociation curve. Thus, although the relative contribution of group III/IV afferents to exercise hyperpnoea was unchanged across environments, the role of this afferent feedback in maintaining $S_{{aO}_{2}}$ and $C_{{aO}_{2}}$ becomes more critical in hypoxia, where equivalent ventilatory suppression leads to a disproportionate decrease in arterial oxygenation. In addition recent evidence has identified a role for group III/IV afferent feedback in supporting gas-exchange efficiency during exercise (Iannetta et al., 2024). We observed a similar widening of the A–aDO_2_ in our fentanyl condition; however this effect was small relative to the independent effects of exercise and hypoxia. Therefore the primary role of group III/IV afferent feedback in protecting arterial oxygenation during exercise appears to be through precise control of exercise hyperpnoea.

### Afferent feedback plays an increasingly important role in maintaining arterial pressure during exercise in hypoxia

Across all environments intrathecal fentanyl elicited a clear decrease in MAP, reaffirming that group III/IV afferent feedback contributes to the maintenance of arterial blood pressure during exercise. Accordingly the reduction in MAP observed with fentanyl is best interpreted as the net haemodynamic consequence of attenuated afferent input, reflecting changes in vascular and central cardiovascular control rather than a direct effect of afferent feedback alone. This blood pressure–lowering response is consistent with evidence that group III/IV afferent feedback accounts for ~50% of the exercise-induced upward resetting of the baroreflex operating point for blood pressure (Hureau et al., 2018). For MAP the effect of intrathecal fentanyl was greater in hypoxia, indicating that group III/IV afferent feedback has a larger contribution to exercising blood pressure in hypoxia. Within this context the greater reduction in MAP likely reflects an increased reliance on afferent-mediated sympathetic vasoconstrictor support to maintain peripheral vascular resistance when oxygen availability is reduced. This observation aligns with the work of Wan et al. (2020b), who showed that suppressing group III/IV afferent feedback during single-leg exercise in acute hypoxia had a greater blood pressure–lowering effect compared to normoxia. The synergistic interaction between the chemoreflex and group III/IV afferent muscle reflex on MAP might be expected to increase with chronic exposure since carotid chemoreceptors increase tonic activity (Getu et al., 2025) and sensitivity (Forster et al., 1971) with prolonged exposure to hypoxia. However, the increased role of group III/IV afferents to exercising MAP was unchanged from acute to chronic exposure. In fact the exaggerated effect of fentanyl in reducing SBP in acute hypoxia is completely eliminated after chronic exposure (Table 2). One possible explanation for this pattern is the reduction in plasma volume that accompanies sustained high-altitude exposure (Roche et al., 2022; Schlittler et al., 2021). Such reductions in central blood volume could plausibly lower venous return and preload, thereby diminishing stroke volume and constraining pulse pressure. In this context the capacity for SBP to decrease further when afferent drive is withdrawn may be limited. Although the mechanisms underlying these differences cannot be resolved with the present data, the consistent blood pressure–lowering response in FENT underscores the role of muscle afferent feedback in supporting arterial pressure when oxygen tension is limited, and highlights the heightened reliance on this feedback during exercise in hypoxia.

Despite this amplified reduction in blood pressure in hypoxia, the influence of group III/IV afferent blockade on HR was modest and did not differ across environments. Due to the decrease in MAP, a compensatory baroreflex-mediated tachycardia might be expected; however HR was only slightly reduced, suggesting that the haemodynamic effects of afferent blockade were not primarily mediated via chronotropic mechanisms. Instead this dissociation between HR and MAP is consistent with a dominant reduction in peripheral vascular resistance, alongside competing influences of reduced sympathetic drive, and maintained central command on cardiac control. This contrasts with the findings of Wan et al. (2020b), who reported a hyper-additive HR response during combined activation of the exercise pressor reflex and hypoxia-induced chemoreflex, interpreted as centrally mediated sympathoexcitation. The absence of a comparable chronotropic interaction here likely reflects differences in experimental context; Wan et al. (2020b) examined reflex co-activation during single-leg knee-extension exercise under severe hypoxaemia, which reflects a much smaller stimulus on the exercise pressor reflex and greater stimulation of the carotid chemoreflex. Therefore the present findings suggest that, under whole-body exercise conditions, afferent blockade primarily alters vascular rather than cardiac components of the pressor response, although the precise mechanisms cannot be resolved from the present data.

### Reflex control of metabolic strain is more critical in acute hypoxia and adapts with prolonged hypoxic exposure

Acute hypoxia imposes a significant metabolic challenge to contracting muscle, constraining oxygen delivery and thereby increasing reliance on anaerobic metabolism (Katz & Sahlin, 1987; Parolin et al., 2000). These findings are well established in both biopsy and P-31 magnetic resonance spectroscopy (^31^P-MRS) studies, which demonstrate greater phosphocreatine depletion, inorganic phosphate accumulation and intracellular acidosis when oxygen availability is limited (Bejder et al., 2025; Haseler et al., 1999; Hogan et al., 1999; Lundby et al., 2009). In the present study this amplified muscle metabolic strain was reflected by higher circulating lactate during acute hypoxia. Intrathecal fentanyl further increased blood lactate and [H^+^] relative to control, and these effects were greater in acute hypoxia than in normoxia, indicating that group III/IV muscle afferents may have contributed more to regulating metabolic strain when oxygen delivery was reduced. Notably, [H^+^] was elevated across all settings, which may reflect fentanyl-induced suppression of ventilation and consequent reductions in CO_2_ clearance (Krapf et al., 1991), whereas increases in lactate more closely tracked the degree of metabolic strain. Prolonged exposure to hypoxia elicits a host of adaptations that collectively improve oxygen transport and lessen the metabolic strain of hypoxic exercise. Increases in $C_{{aO}_{2}}$ (Bärtsch & Saltin, 2008; Siebenmann et al., 2024), together with improved acid–base regulation (Juel et al., 2003) and enhanced muscle buffering capacity (Mizuno et al., 2008), enhance oxygen utilisation and help delay intramuscular acidosis during exercise (Bender et al., 1989). Relative to acute exposure, acclimatisation to hypoxia may reduce muscle lactate accumulation at a given workload by lowering glycolytic flux and enhancing oxidative phosphorylation (Green et al., 1992), though this response is not consistently observed (van Hall et al., 2009). Nevertheless, in the present study acclimatisation partly restored sea-level $C_{{aO}_{2}}$ and was accompanied by smaller increases in blood lactate and [H^+^] during matched-workload exercise in hypoxia. Given that group III/IV afferents are sensitive to intramuscular lactate concentrations and [H^+^] (Jankowski et al., 2013; Light et al., 2008), the smaller fentanyl-associated perturbations in blood lactate and [H^+^] after acclimatisation are consistent with a reduced metabolic stimulus for afferent activation when oxygen transport is reestablished.

### Group III/IV afferents contribute to breathing pattern adaptation after acclimatisation without alleviating dyspnoea

At matched absolute workloads ratings of dyspnoea and leg discomfort were 60%–70% higher in acute hypoxia and did not improve with acclimatisation. These observations provide insight into the mechanisms underlying perceptual strain in hypoxia. Despite acclimatisation increasing exercising ventilation by 22%, achieved through greater breathing frequency and a tidal volume reduced to ~60% of normoxic values, ratings of dyspnoea remained unchanged between acute and chronic hypoxia. Given that large intrapleural pressure swings contribute to dyspnoeic sensations (Gorini et al., 1996), this rapid, shallow breathing pattern may act to minimise perceptual strain when the overall ventilatory demand is high. Notably, the influence of group III/IV afferent activity on breathing frequency, rather than tidal volume, was enhanced in acute hypoxia and further amplified after acclimatisation. These findings suggest that group III/IV afferent feedback contributes to the development of the rapid, shallow breathing pattern that is naturally adopted during exercise in hypoxia.

### Methodological considerations

Several methodological factors should be acknowledged when interpreting these findings. Intrathecal fentanyl only partially blocks group III/IV afferent feedback; it has been estimated that ~50% of this feedback remains intact (Hureau et al., 2018). As such, the contribution of these afferents to the cardiorespiratory and perceptual responses observed here is likely underestimated. Although a number of observations argue against cephalad migration of fentanyl (including the absence of resting ventilatory or cardiovascular effects, and the lack of suppression of ${\dot{V}}_{E},\;P_{{aCO}_{2}},\;{\dot{V}}_{E}/{VCO}_{2}$ or HR during the arm-crank control experiment), the approach used to exclude central effects was indirect. Arterial pressure measurements obtained during the arm-crank control condition were not interpretable due to movement artefact, preventing direct assessment of blood pressure regulation in this setting. Consequently subtle central cardiovascular influences during exercise cannot be definitively excluded. Beyond the pharmacological considerations associated with intrathecal fentanyl, sea-level and high-altitude trials were separated by ~3 months and conducted in a fixed order due to the logistical constraints of the high-altitude expedition. However, the primary comparisons (fentanyl *vs*. control) were performed within setting and were randomised and blinded, minimising the potential influence of these factors on the key outcomes. Additionally, the 7- to 9-day altitude acclimatisation period represents an early stage of adaptation; with longer residence, continued haematological (Bärtsch & Saltin, 2008) and muscular adjustments (Mizuno et al., 2008) could further refine afferent control. Although systemic markers such as blood lactate and pH provide a general index of muscle metabolic strain, they do not fully capture the intramuscular milieu that governs group III/IV afferent activation (Blain et al., 2016). The absence of direct assessment of intramuscular metabolites therefore limits mechanistic interpretation of the afferent-mediated responses observed here. Finally, the absence of direct measures of leg blood flow or oxygen delivery represents an important limitation, particularly due to the altered haemodynamic environment of hypoxia. Without concurrent measures of local perfusion, it remains uncertain whether differences in oxygen delivery or metabolic stability primarily underlie the observed changes in afferent contribution.

### Summary and conclusion

Group III/IV muscle afferent feedback plays a critical role in regulating ventilatory and pressor responses during steady-state exercise in normoxic and hypoxic settings. The importance of this feedback is accentuated in hypoxia, where small perturbations in ventilatory control and perfusion pressure have amplified consequences for oxygen transport when oxygen availability is limited. Accordingly, intrathecal fentanyl–induced suppression of exercise hyperpnoea resulted in larger reductions in arterial oxygen saturation and oxygen content, alongside a greater blood pressure–lowering response in hypoxia. In contrast, the contribution of group III/IV afferents to circulating lactate and hydrogen ion concentration was accentuated during acute hypoxia and attenuated after acclimatisation, indicating that muscle reflex control of metabolic strain diminishes as oxygen transport is partially restored. Together these findings identify group III/IV muscle afferent feedback as an adaptive control mechanism that is essential for maintaining arterial oxygenation and blood pressure during exercise in hypoxia.

## Supplementary Material

Supplementary material

Supporting information

Additional supporting information can be found online in the Supporting Information section at the end of the HTML view of the article. Supporting information files available:

Peer Review History

The peer review history is available in the Supporting Information section of this article (https://doi.org/10.1113/JP290914#support-information-section).

## Figures and Tables

**Table 1.** Ventilatory and gas-exchange responses under control and fentanyl conditions across environments and exercise intensities

		SL normoxia		SL hypoxia		HA hypoxia		Main effects				Two-way interactions	

		CON	FENT	CON	FENT	CON	FENT	Intensity	P_I_O_2_	Acclimatisation	Condition	Cond×P_I_O_2_	Cond×acclimatisation

**N**		14	14	14	14	17	17						
**P_I_O_2_ (mmHg)**	Rest	139 ± 1	139 ± 1	90 ± 1	89 ± 2	91 ± 1	91 ± 1	<0.001	<0.001	<0.001	0.461	0.997	0.916
	Ex1	139 ± 1	139 ± 2	90 ± 0	89 ± 1	91 ± 1	91 ± 1						
	Ex2	139 ± 1	139 ± 2	90 ± 0	90 ± 2	91 ± 1	92 ± 2						
	Ex3	139 ± 1	139 ± 2	96 ± 1	96 ± 1	100 ± 1	100 ± 1						
${\dot{V}}_{E}$ **(L·min^−1^)**	Rest	17 ± 2	17 ± 3	25 ± 5	25 ± 4	30 ± 8	29 ± 7	<0.001	<0.001	<0.001	<0.001	0.859	0.148
	Ex1	38 ± 5	36 ± 5	51 ± 9	48 ± 8	60 ± 13	56 ± 10						
	Ex2	51 ± 7	47 ± 8	69 ± 11	66 ± 12	84 ± 18	78 ± 14						
	Ex3	66 ± 12	62 ± 11	96 ± 21	94 ± 20	119 ± 28	108 ± 22						
**Fb (bpm)**	Rest	18 ± 3	16 ± 4	18 ± 4	18 ± 4	25 ± 3	22 ± 6	<0.001	<0.001	<0.001	<0.001	0.796	0.008
	Ex1	21 ± 4	19 ± 4	26 ± 5	25 ± 6	33 ± 5	30 ± 5						
	Ex2	23 ± 4	21 ± 4	31 ± 5	30 ± 7	39 ± 6	35 ± 7						
	Ex3	27 ± 4	26 ± 6	38 ± 7	36 ± 7	49 ± 9	42 ± 7						
$\dot{V}T$ **(L)**	Rest	0.8 ± 0.2	0.9 ± 0.4	1.2 ± 0.4	1.2 ± 0.4	0.7 ± 0.2	0.7 ± 0.2	<0.001	<0.001	<0.001	<0.001	0.920	0.701
	Ex1	1.6 ± 0.4	1.6 ± 0.5	1.7 ± 0.4	1.8 ± 0.5	1.0 ± 0.2	1.1 ± 0.2						
	Ex2	1.9 ± 0.4	2.0 ± 0.5	1.9 ± 0.4	2.0 ± 0.5	1.2 ± 0.2	1.2 ± 0.3						
	Ex3	2.1 ± 0.4	2.1 ± 0.5	2.1 ± 0.4	2.3 ± 0.5	1.3 ± 0.3	1.4 ± 0.3						
${\dot{V}O}_{2}$ **(L·min^−1^)**	Rest	0.6 ± 0.1	0.7 ± 0.2	0.6 ± 0.2	0.6 ± 0.1	0.6 ± 0.2	0.6 ± 0.2	<0.001	<0.001	0.957	0.384	0.636	0.371
	Ex1	1.7 ± 0.2	1.7 ± 0.2	1.5 ± 0.3	1.6 ± 0.2	1.5 ± 0.3	1.5 ± 0.3						
	Ex2	2.1 ± 0.3	2.1 ± 0.3	2.0 ± 0.4	2.0 ± 0.3	2.0 ± 0.4	2.0 ± 0.4						
	Ex3	2.6 ± 0.4	2.6 ± 0.4	2.3 ± 0.4	2.4 ± 0.3	2.4 ± 0.4	2.4 ± 0.4						
${\dot{V}}_{{CO}_{2}}$ **(L·min^−1^)**	Rest	0.5 ± 0.1	0.5 ± 0.1	0.7 ± 0.2	0.8 ± 0.2	0.7 ± 0.2	0.7 ± 0.2	<0.001	<0.001	<0.001	0.090	0.473	0.805
	Ex1	1.4 ± 0.2	1.4 ± 0.2	1.6 ± 0.3	1.6 ± 0.2	1.4 ± 0.3	1.5 ± 0.3						
	Ex2	1.9 ± 0.3	1.9 ± 0.3	2.1 ± 0.3	2.2 ± 0.3	2.0 ± 0.4	2.0 ± 0.4						
	Ex3	2.6 ± 0.4	2.6 ± 0.4	2.8 ± 0.4	3.0 ± 0.5	2.6 ± 0.5	2.7 ± 0.4						
**RER**	Rest	0.81 ± 0.05	0.79 ± 0.05	1.34 ± 0.23	1.32 ± 0.22	1.15 ± 0.10	1.17 ± 0.12	<0.001	<0.001	<0.001	0.936	0.799	0.191
	Ex1	0.83 ± 0.05	0.81 ± 0.02	1.04 ± 0.12	1.02 ± 0.08	0.95 ± 0.05	0.95 ± 0.08						
	Ex2	0.92 ± 0.04	0.91 ± 0.03	1.11 ± 0.14	1.10 ± 0.07	1.00 ± 0.06	1.02 ± 0.07						
	Ex3	0.99 ± 0.04	1.00 ± 0.03	1.26 ± 0.16	1.24 ± 0.09	1.10 ± 0.08	1.13 ± 0.09						
${\dot{V}}_{E}/{\dot{V}}_{{CO}_{2}}$	Rest	0.37 ± 0.05	0.34 ± 0.04	0.34 ± 0.02	0.33 ± 0.04	0.47 ± 0.06	0.44 ± 0.07	<0.001	<0.001	<0.001	<0.001	0.765	0.104
	Ex1	0.28 ± 0.01	0.26 ± 0.02	0.32 ± 0.03	0.31 ± 0.03	0.42 ± 0.05	0.39 ± 0.04						
	Ex2	0.26 ± 0.02	0.24 ± 0.03	0.32 ± 0.03	0.31 ± 0.04	0.42 ± 0.05	0.39 ± 0.05						
	Ex3	0.26 ± 0.02	0.24 ± 0.03	0.34 ± 0.04	0.32 ± 0.04	0.45 ± 0.07	0.40 ± 0.06						
$P_{{AO}_{2}}$ **(mmHg)**	Rest	95 ± 3	93 ± 5	62 ± 4	60 ± 5	67 ± 3	66 ± 3	<0.001	<0.001	<0.001	<0.001	0.424	0.410
	Ex1	94 ± 4	90 ± 5	56 ± 4	54 ± 3	63 ± 3	61 ± 2						
	Ex2	96 ± 3	92 ± 5	58 ± 4	57 ± 4	65 ± 3	64 ± 3						
	Ex3	98 ± 4	96 ± 6	69 ± 4	67 ± 4	77 ± 4	76 ± 2						
$P_{{ETO}_{2}}$ **(mmHg)**	Rest	101 ± 3	99 ± 5	63 ± 4	62 ± 5	71 ± 2	69 ± 3	0.005	<0.001	<0.001	<0.001	0.408	0.983
	Ex1	93 ± 4	90 ± 5	56 ± 3	53 ± 3	65 ± 3	63 ± 2						
	Ex2	95 ± 4	91 ± 6	57 ± 3	55 ± 4	66 ± 3	64 ± 3						
	Ex3	96 ± 5	94 ± 7	61 ± 4	60 ± 4	70 ± 4	67 ± 3						
$P_{ETCO_{2}}$ **(mmHg)**	Rest	35 ± 2	36 ± 2	34 ± 2	36 ± 4	24 ± 6	27 ± 3	<0.001	<0.001	<0.001	<0.001	0.586	0.128
	Ex1	40 ± 2	43 ± 3	35 ± 2	37 ± 3	26 ± 6	29 ± 3						
	Ex2	43 ± 3	46 ± 5	35 ± 3	38 ± 4	25 ± 6	29 ± 3						
	Ex3	44 ± 4	47 ± 6	34 ± 4	36 ± 5	24 ± 7	28 ± 4						
$P_{{aO}_{2}}$ **(mmHg)**	Rest	92.4 ± 4.1	91.9 ± 7.3	59.0 ± 5.5	56.7 ± 5.8	65.5 ± 3.4	63.8 ± 2.7	<0.001	<0.001	<0.001	<0.001	0.144	0.809
	Ex1	89.1 ± 5.0	82.9 ± 6.9	43.3 ± 4.0	40.4 ± 4.3	52.2 ± 4.6	49.9 ± 3.1						
	Ex2	84.2 ± 5.9	78.2 ± 7.0	40.6 ± 4.1	39.4 ± 4.5	49.0 ± 5.0	47.2 ± 3.3						
	Ex3	80.0 ± 6.9	77.2 ± 10.0	41.5 ± 3.7	40.7 ± 3.5	48.6 ± 4.8	47.4 ± 3.6						
$P_{{aCO}_{2}}$ **(mmHg)**	Rest	37.6 ± 1.8	38.2 ± 3.1	35.2 ± 2.0	37.0 ± 3.7	26.8 ± 2.8	27.9 ± 2.2	<0.001	<0.001	<0.001	<0.001	0.589	0.511
	Ex1	39.5 ± 2.7	42.2 ± 3.7	34.1 ± 2.1	36.0 ± 3.0	27.2 ± 2.5	28.7 ± 2.2						
	Ex2	41.1 ± 2.4	44.2 ± 3.6	33.9 ± 2.2	36.1 ± 3.9	26.4 ± 2.5	28.2 ± 2.0						
	Ex3	41.1 ± 3.0	44.0 ± 4.5	32.7 ± 2.9	34.6 ± 4.4	24.8 ± 2.9	26.7 ± 2.4						
**A–aDO_2_ (mmHg)**	Rest	1.7 ± 2.4	0.4 ± 3.5	2.5 ± 2.3	3.1 ± 1.9	1.6 ± 2.1	2.3 ± 1.9	<0.001	<0.001	0.180	0.013	0.186	0.399
	Ex1	3.9 ± 2.1	6.2 ± 2.2	13.0 ± 2.2	13.5 ± 2.7	10.4 ± 2.6	11.3 ± 2.8						
	Ex2	11.0 ± 2.9	13.4 ± 4.1	17.7 ± 2.4	17.0 ± 2.7	15.5 ± 3.2	16.4 ± 3.2						
	Ex3	17.5 ± 4.4	17.9 ± 5.7	27.1 ± 2.5	25.6 ± 2.7	28.7 ± 3.0	28.7 ± 2.9						
$S_{{aO}_{2}}$ **(%)**	Rest	97.8 ± 0.5	97.6 ± 0.9	90.2 ± 2.4	87.7 ± 3.1	91.9 ± 1.6	90.7 ± 1.3	<0.001	<0.001	<0.001	<0.001	0.007	0.173
	Ex1	97.4 ± 0.6	96.2 ± 1.5	79.6 ± 4.1	74.5 ± 5.8	85.1 ± 3.6	82.4 ± 3.3						
	Ex2	96.4 ± 1.1	94.9 ± 1.8	75.3 ± 5.9	71.1 ± 7.0	82.4 ± 4.8	79.4 ± 4.0						
	Ex3	95.3 ± 1.6	93.8 ± 3.0	72.7 ± 6.6	68.8 ± 7.0	79.8 ± 5.4	76.8 ± 4.6						
$C_{{aO}_{2}}$ **(mL·dL^−1^)**	Rest	20.1 ± 2.0	20.1 ± 1.7	18.6 ± 1.9	18.1 ± 2.1	19.8 ± 2.1	19.4 ± 1.6	<0.001	<0.001	<0.001	<0.001	0.016	0.832
	Ex1	20.1 ± 1.9	19.9 ± 1.8	16.3 ± 2.0	15.3 ± 2.0	18.2 ± 2.2	17.5 ± 1.8						
	Ex2	20.0 ± 2.0	19.7 ± 1.8	15.7 ± 2.1	14.7 ± 1.9	17.7 ± 2.3	17.0 ± 1.7						
	Ex3	19.9 ± 2.0	19.7 ± 2.0	15.5 ± 2.2	14.8 ± 2.0	17.5 ± 2.3	16.8 ± 1.7						

Data are presented as mean ± SD for each setting: sea-level normoxia (SL normoxia), sea-level hypoxia (SL hypoxia) and high-altitude hypoxia (HA hypoxia), under control (CON) and fentanyl (FENT) conditions. Exercise stages include rest and three workloads (Ex1–Ex3). Main effects of exercise intensity, inspired oxygen partial pressure (P_I_O_2_, normoxia *vs*. hypoxia), acclimatisation (SL *vs*. HA), condition (CON *vs*. FENT) and key interactions (*condition* × *P_I_O_2_* and *condition* × *acclimatisation*) are reported (*P*-values).

Abbreviations: A–aDO_2_, alveolar-arterial O_2_ difference; $C_{{aO}_{2}}$, arterial oxygen content; Fb, breathing frequency; $P_{{AO}_{2}}$, alveolar oxygen tension; $P_{{aO}_{2}}/P_{{aCO}_{2}}$, arterial oxygen/CO_2_ tension; $P_{{ETO}_{2}}/P_{{ETCO}_{2}}$, end-tidal O_2_/CO_2_; P_I_O_2_, inspired oxygen partial pressure; RER, respiratory exchange ratio; $S_{{aO}_{2}}$, arterial oxygen saturation; ${\dot{V}}_{{CO}_{2}}$, carbon dioxide output; ${\dot{V}}_{E}$, minute ventilation; ${\dot{V}}_{E}/{\dot{V}}_{{CO}_{2}}$, ventilatory equivalent for CO_2_; ${\dot{VO}}_{2}$, oxygen uptake; VT, tidal volume;.

**Table 2.** Cardiovascular responses under control and fentanyl conditions across environments and exercise intensities

		SL normoxia		SL hypoxia		HA hypoxia		Main effects				Two-way interactions	

		CON	FENT	CON	FENT	CON	FENT	Intensity	P_I_O_2_	Acclimatisation	Condition	Cond×P_I_O_2_	Cond×acclimatisation

**N**		14	14	14	14	17	17						
**HR (bpm)**	Rest	85 ± 13	85 ± 15	113 ± 11	109 ± 18	116 ± 12	115 ± 16	<0.001	<0.001	0.003	<0.001	0.339	0.556
	Ex1	119 ± 10	113 ± 12	145 ± 13	142 ± 16	137 ± 10	135 ± 13						
	Ex2	139 ± 11	130 ± 13	161 ± 13	155 ± 17	153 ± 8	149 ± 13						
	Ex3	157 ± 12	150 ± 15	173 ± 10	173 ± 10	164 ± 9	162 ± 11						
**MAP (mmHg)**	Rest	107 ± 10	108 ± 9	110 ± 11	105 ± 10	111 ± 12	106 ± 13	<0.001	<0.001	0.009	<0.001	0.021	0.702
	Ex1	113 ± 11	108 ± 8	111 ± 13	100 ± 10	108 ± 11	102 ± 12						
	Ex2	116 ± 11	107 ± 8	113 ± 12	98 ± 14	112 ± 12	102 ± 11						
	Ex3	122 ± 11	111 ± 8	118 ± 12	105 ± 10	117 ± 12	104 ± 10						
**SBP (mmHg)**	Rest	151 ± 14	154 ± 14	164 ± 15	157 ± 12	164 ± 14	162 ± 14	<0.001	0.502	0.073	<0.001	0.031	<0.001
	Ex1	182 ± 17	173 ± 15	189 ± 21	170 ± 20	177 ± 19	179 ± 20						
	Ex2	197 ± 16	182 ± 19	201 ± 24	175 ± 30	192 ± 25	191 ± 23						
	Ex3	212 ± 21	196 ± 21	212 ± 25	189 ± 29	207 ± 28	199 ± 25						
**DBP (mmHg)**	Rest	83 ± 8	83 ± 8	85 ± 10	81 ± 9	89 ± 12	83 ± 13	<0.001	<0.001	<0.001	<0.001	0.037	0.087
	Ex1	79 ± 9	76 ± 7	78 ± 11	70 ± 9	78 ± 9	70 ± 10						
	Ex2	79 ± 9	73 ± 7	76 ± 10	66 ± 11	78 ± 10	68 ± 9						
	Ex3	81 ± 10	74 ± 8	76 ± 10	69 ± 8	78 ± 10	66 ± 8						

Data are presented as mean ± SD for each setting: sea-level normoxia (SL normoxia), sea-level hypoxia (SL hypoxia) and high-altitude hypoxia (HA hypoxia), under control (CON) and fentanyl (FENT) conditions. Exercise stages include rest and three workloads (Ex1–Ex3). Main effects of exercise intensity, inspired oxygen partial pressure (P_I_O_2_, normoxia *vs*. hypoxia), acclimatisation (SL *vs*. HA), condition (CON *vs*. FENT) and key interactions (condition × P_I_O_2_ and condition × acclimatisation) are reported (*P*-values).

Abbreviations: DBP, diastolic blood pressure; HR, heart rate; MAP, mean arterial pressure; SBP, systolic blood pressure.

**Table 3.** Acid-base, metabolic, haematological and core temperature responses under control and fentanyl conditions across environments and exercise intensities

		SL normoxia		SL hypoxia		HA hypoxia		Main effects				Two-way interactions	

		CON	FENT	CON	FENT	CON	FENT	Intensity	P_I_O_2_	Acclimatisation	Condition	Cond×P_I_O_2_	Cond×acclimatisation

**N**		14	14	14	14	17	17						
**PH**	Rest	7.41 ± 0.02	7.40 ± 0.02	7.39 ± 0.03	7.37 ± 0.03	7.40 ± 0.02	7.38 ± 0.03	<0.001	0.017	<0.001	<0.001	0.026	0.098
	Ex1	7.39 ± 0.02	7.37 ± 0.02	7.43 ± 0.02	7.40 ± 0.03	7.42 ± 0.02	7.41 ± 0.02						
	Ex2	7.37 ± 0.02	7.35 ± 0.03	7.41 ± 0.03	7.37 ± 0.05	7.42 ± 0.02	7.40 ± 0.02						
	Ex3	7.35 ± 0.03	7.32 ± 0.04	7.34 ± 0.04	7.30 ± 0.07	7.38 ± 0.03	7.36 ± 0.03						
**[H^+^] (nmol·L^−1^)**	Rest	38.8 ± 1.6	39.1 ± 1.5	39.5 ± 2.6	42.0 ± 3.2	39.2 ± 2.0	40.4 ± 2.8	<0.001	0.001	<0.001	<0.001	0.034	0.023
	Ex1	40.0 ± 1.7	41.7 ± 2.5	36.5 ± 1.6	38.4 ± 2.9	37.0 ± 2.1	38.2 ± 2.1						
	Ex2	41.9 ± 2.2	44.0 ± 3.1	37.7 ± 2.5	41.1 ± 4.7	36.6 ± 1.9	38.4 ± 2.1						
	Ex3	44.2 ± 2.9	46.8 ± 4.3	44.0 ± 4.2	48.9 ± 8.2	40.6 ± 2.5	42.6 ± 3.1						
**[HCO_3_^−^] (mequiv L^−1^)**	Rest	23.0 ± 1.2	23.2 ± 1.4	21.0 ± 1.6	20.7 ± 2.1	16.2 ± 1.8	16.3 ± 1.7	<0.001	<0.001	<0.001	0.578	0.034	0.100
	Ex1	23.4 ± 1.3	24.0 ± 1.4	22.0 ± 1.7	22.0 ± 1.9	17.4 ± 1.8	17.7 ± 1.5						
	Ex2	23.2 ± 1.3	23.7 ± 1.4	21.1 ± 1.4	20.6 ± 2.2	17.0 ± 1.6	17.2 ± 1.2						
	Ex3	21.9 ± 1.6	22.0 ± 2.0	17.3 ± 1.6	16.6 ± 2.2	14.3 ± 1.8	14.6 ± 1.5						
**[La^−^] (mmol·L^−1^)**	Rest	1.5 ± 0.5	1.7 ± 0.5	2.8 ± 1.1	3.7 ± 1.2	3.5 ± 1.5	3.5 ± 1.7	<0.001	<0.001	<0.001	<0.001	0.012	0.002
	Ex1	1.5 ± 0.4	1.7 ± 0.4	2.4 ± 0.9	3.0 ± 1.1	2.8 ± 1.4	2.8 ± 1.5						
	Ex2	2.2 ± 0.7	2.3 ± 0.7	4.0 ± 1.1	5.3 ± 1.9	3.6 ± 1.3	3.8 ± 1.4						
	Ex3	3.8 ± 1.3	4.5 ± 1.2	9.0 ± 2.0	11.0 ± 3.0	7.4 ± 1.9	7.7 ± 2.2						
**[Glu] (mmol·L^−1^)**	Rest	5.5 ± 0.4	5.7 ± 0.6	5.4 ± 0.3	5.6 ± 0.4	5.9 ± 0.4	5.7 ± 0.3	0.001	0.129	0.087	0.026	0.350	0.011
	Ex1	5.5 ± 0.4	5.7 ± 0.6	5.4 ± 0.3	5.5 ± 0.4	5.6 ± 0.4	5.6 ± 0.3						
	Ex2	5.4 ± 0.3	5.7 ± 0.6	5.3 ± 0.3	5.5 ± 0.3	5.5 ± 0.5	5.5 ± 0.4						
	Ex3	5.4 ± 0.3	5.7 ± 0.5	5.4 ± 0.4	5.7 ± 0.4	5.5 ± 0.6	5.4 ± 0.4						
**Het (%)**	Rest	46.3 ± 4.7	46.5 ± 4.3	46.7 ± 4.8	46.7 ± 4.5	48.7 ± 5.1	48.3 ± 4.2	<0.001	0.037	<0.001	0.159	0.577	0.018
	Ex1	46.5 ± 4.6	46.8 ± 4.3	46.5 ± 4.8	46.6 ± 4.2	48.5 ± 5.2	48.1 ± 4.2						
	Ex2	46.9 ± 4.6	46.8 ± 4.3	47.3 ± 4.7	46.9 ± 4.4	48.7 ± 5.1	48.5 ± 4.0						
	Ex3	47.3 ± 4.8	47.4 ± 4.5	48.3 ± 4.6	48.8 ± 4.5	49.6 ± 5.1	49.5 ± 4.1						
**[Hb] (g·dL^−1^)**	Rest	15.1 ± 1.5	15.2 ± 1.4	15.2 ± 1.5	15.2 ± 1.5	15.9 ± 1.7	15.8 ± 1.4	<0.001	0.041	<0.001	0.167	0.654	0.016
	Ex1	15.2 ± 1.5	15.3 ± 1.4	15.2 ± 1.6	15.2 ± 1.4	15.8 ± 1.7	15.7 ± 1.4						
	Ex2	15.3 ± 1.5	15.3 ± 1.4	15.4 ± 1.5	15.3 ± 1.4	15.9 ± 1.7	15.8 ± 1.3						
	Ex3	15.4 ± 1.6	15.5 ± 1.5	15.7 ± 1.5	15.9 ± 1.5	16.2 ± 1.7	16.1 ± 1.3						
**TREC (°C)**	Rest	37.5 ± 0.3	37.5 ± 0.3	37.8 ± 0.3	37.9 ± 0.2	37.6 ± 0.2	37.7 ± 0.4	<0.001	<0.001	<0.001	<0.001	0.087	0.734
	Ex1	37.5 ± 0.3	37.5 ± 0.3	37.8 ± 0.3	37.9 ± 0.2	37.7 ± 0.2	37.8 ± 0.4						
	Ex2	37.6 ± 0.3	37.6 ± 0.3	37.9 ± 0.3	38.0 ± 0.2	37.8 ± 0.2	37.9 ± 0.4						
	Ex3	37.7 ± 0.3	37.7 ± 0.2	38.1 ± 0.3	38.2 ± 0.2	37.9 ± 0.3	38.0 ± 0.3						

Data are presented as mean ± SD for each setting: sea-level normoxia (SL normoxia), sea-level hypoxia (SL hypoxia) and high-altitude hypoxia (HA hypoxia), under control (CON) and fentanyl (FENT) conditions. Exercise stages include rest and three workloads (Ex1–Ex3). Main effects of exercise intensity, inspired oxygen partial pressure (P_I_O_2_, normoxia *vs*. hypoxia), acclimatisation (SL *vs*. HA), condition (CON *vs*. FENT) and key interactions (*condition* × *P_I_O_2_* and *condition* × *acclimatisation*) are reported (*P*-values).

Abbreviations: [Glu], blood glucose concentration; [H^+^], hydrogen ion concentration; [Hb], haemoglobin concentration; [HCO_3_^−^], bicarbonate concentration; Hct, haematocrit; [La^−^], blood lactate concentration; pH, blood pH; TREC, rectal temperature.

**Table 4.** Perceptual responses under control and fentanyl conditions across environments and exercise intensities

		SL normoxia		SL hypoxia		HA hypoxia		Main effects				Two-way interactions	

		CON	FENT	CON	FENT	CON	FENT	Intensity	P_I_O_2_	Acclimatisation	Condition	Cond×P_I_O_2_	Cond×acclimatisation

**N**		14	14	14	14	17	17						
**Dyspnoea (0–10)**	Rest							<0.001	<0.001	0.022	<0.001	0.496	0.299
	Ex1	0.9 ± 0.7	0.5 ± 0.5	1.6 ± 1.0	1.3 ± 0.9	1.2 ± 0.8	1.0 ± 0.6						
	Ex2	2.2 ± 0.8	1.7 ± 0.9	3.6 ± 0.9	3.0 ± 1.2	3.0 ± 0.9	2.5 ± 0.9						
	Ex3	3.4 ± 1.0	2.7 ± 0.9	5.4 ± 1.4	5.0 ± 1.4	5.8 ± 1.8	4.4 ± 1.3						
**Leg discomfort (0–10)**	Rest							<0.001	<0.001	0.022	<0.001	0.963	0.982
	Ex1	0.5 ± 0.9	0.3 ± 0.6	0.9 ± 0.7	0.6 ± 0.6	0.6 ± 0.5	0.5 ± 0.5						
	Ex2	1.4 ± 0.9	0.8 ± 0.8	2.5 ± 1.2	1.9 ± 1.2	2.1 ± 1.0	1.4 ± 1.0						
	Ex3	2.6 ± 0.8	1.5 ± 1.0	4.3 ± 1.2	3.2 ± 1.6	4.5 ± 1.9	3.3 ± 1.8						

Data are presented as mean ± SD for each setting: sea-level normoxia (SL normoxia), sea-level hypoxia (SL hypoxia) and high-altitude hypoxia (HA hypoxia), under control (CON) and fentanyl (FENT) conditions. Exercise stages include rest and three workloads (Ex1–Ex3). Main effects of exercise intensity, inspired oxygen partial pressure (P_I_O_2_, normoxia *vs*. hypoxia), acclimatisation (SL *vs*. HA), condition (CON *vs*. FENT) and key interactions (*condition* × *P_I_O_2_* and *condition* × *acclimatisation*) are reported (*P*-values).

## Data Availability

The datasets generated and analysed during the current study are available from the corresponding author on reasonable request.

## Key points

- Group III/IV muscle afferent feedback contributes to normal exercise hyperpnoea and blood pressure regulation; whether its functional contribution is altered by acute and chronic hypoxia is unknown.
- In a double-blind, placebo-controlled cross-over design, 14 participants completed steady-state cycling with intact (placebo) or attenuated (intrathecal fentanyl) group III/IV afferent feedback in normoxia, acute hypoxia and after 7–9 days of acclimatisation at 3800 m.
- Intrathecal fentanyl suppressed exercise hyperpnoea similarly across environments but caused exaggerated arterial oxygen desaturation during hypoxic exercise, reflecting operation on the steep portion of the oxygen–haemoglobin dissociation curve.
- The blood pressure–lowering effect of intrathecal fentanyl was greater during hypoxic exercise; effects on metabolic strain were greatest in acute hypoxia and largely normalised after acclimatisation.
- These findings highlight the functional importance of group III/IV muscle afferent feedback during hypoxic exercise, where small perturbations in ventilatory and pressor control have amplified consequences for oxygen transport.

## References

[R1] Amann M, Blain GM, Proctor LT, Sebranek JJ, Pegelow DF, & Dempsey JA (2010). Group III and IV muscle afferents contribute to ventilatory and cardiovascular response to rhythmic exercise in humans. Journal of Applied Physiology, 109(4), 966–976. 10.1152/japplphysiol.00462.2010 PMC2963332 20634355

[R2] Amann M, Goodall S, Twomey R, Subudhi AW, Lovering AT, & Roach RC (2013). AltitudeOmics: On the consequences of high-altitude acclimatization for the development of fatigue during locomotor exercise in humans. Journal of Applied Physiology, 115(5), 634–642. 10.1152/japplphysiol.00606.2013 PMC3763067 23813531

[R3] Amann M, & Kayser B (2009). Nervous system function during exercise in hypoxia. High Altitude Medicine & Biology, 10(2), 149–164. 10.1089/ham.2008.1105 19555297

[R4] Amann M, Proctor LT, Sebranek JJ, Pegelow DF, & Dempsey JA (2009). Opioid-mediated muscle afferents inhibit central motor drive and limit peripheral muscle fatigue development in humans. The Journal of Physiology, 587(1), 271–283. 10.1113/jphysiol.2008.163303 PMC2670040 19015193

[R5] Bärtsch P, & Saltin B (2008). General introduction to altitude adaptation and mountain sickness. Scandinavian Journal of Medicine & Science in Sports, 18(Suppl 1), 1–10. 10.1111/j.1600-0838.2008.00827.x 18665947

[R6] Bejder J, Graae J, Andersen JB, Barbieri RA, Campos EZ, Bangsbo J, Nybo L, & Nordsborg NB (2025). Time-course of muscle fatigue development during intense exercise in hypoxia and normoxia. Scientific Reports, 15(1), 14065. 10.1038/s41598-025-98762-x PMC12018925 40269052

[R7] Bender PR, Groves BM, McCullough RE, McCullough RG, Trad L, Young AJ, Cymerman A, & Reeves JT (1989). Decreased exercise muscle lactate release after high altitude acclimatization. Journal of Applied Physiology, 67(4), 1456–1462. 10.1152/jappl.1989.67.4.1456 2793749

[R8] Blain GM, Mangum TS, Sidhu SK, Weavil JC, Hureau TJ, Jessop JE, Bledsoe AD, Richardson RS, & Amann M (2016). Group III/IV muscle afferents limit the intramuscular metabolic perturbation during whole body exercise in humans. The Journal of Physiology, 594(18), 5303–5315. 10.1113/JP272283 PMC5023715 27241818

[R9] Borg G (1998). Borg’s perceived exertion and pain scales. Human Kinetics.

[R10] Chronos N, Adams L, & Guz A (1988). Effect of hyperoxia and hypoxia on exercise-induced breathlessness in normal subjects. Clinical Science, 74(5), 531–537. 10.1042/cs0740531 3370920

[R11] Dempsey JA, Reddan WG, Birnbaum ML, Forster HV, Thoden JS, Grover RF, & Rankin J (1971). Effects of acute through life-long hypoxic exposure on exercise pulmonary gas exchange. Respiration Physiology, 13(1), 62–89. 10.1016/0034-5687(71)90065-x 5112830

[R12] Dousset E, Decherchi P, Grelot L, & Jammes Y (2001). Effects of chronic hypoxemia on the afferent nerve activities from skeletal muscle. American Journal of Respiratory and Critical Care Medicine, 164(8), 1476–1480. 10.1164/ajrccm.164.8.2010135 11704599

[R13] Forster HV, Dempsey JA, Birnbaum ML, Reddan WG, Thoden J, Grover RF, & Rankin J (1971). Effect of chronic exposure to hypoxia on ventilatory response to CO_2_ and hypoxia. Journal of Applied Physiology, 31(4), 586–592. 10.1152/jappl.1971.31.4.586 5111007

[R14] Gagnon P, Bussières JS, Ribeiro F, Gagnon SL, Saey D, Gagné N, Provencher S, & Maltais F (2012). Influences of spinal anesthesia on exercise tolerance in patients with chronic obstructive pulmonary disease. American Journal of Respiratory and Critical Care Medicine, 186(7), 606–615. 10.1164/rccm.201203-0404OC 22822019

[R15] Getu AA, Brewster LM, Gibbons TD, Anholm JD, Stembridge M, Ainslie PN, & Carr JMJR (2025). Elevated carotid body tonic activity contributes to ventilatory acclimatization and de-acclimatization to high altitude at rest and during exercise. The Journal of Physiology. Advance online publication. 10.1113/JP289355 41014645

[R16] Gorini M, Misuri G, Corrado A, Duranti R, Iandelli I, De Paola E, & Scano G (1996). Breathing pattern and carbon dioxide retention in severe chronic obstructive pulmonary disease. Thorax, 51(7), 677–683. 10.1136/thx.51.7.677 PMC472488 8882072

[R17] Green HJ, Sutton JR, Wolfel EE, Reeves JT, Butterfield GE, & Brooks GA (1992). Altitude acclimatization and energy metabolic adaptations in skeletal muscle during exercise. Journal of Applied Physiology, 73(6), 2701–2708. 10.1152/jappl.1992.73.6.2701 1490988

[R18] Haseler LJ, Hogan MC, & Richardson RS (1999). Skeletal muscle phosphocreatine recovery in exercise-trained humans is dependent on O_2_ availability. Journal of Applied Physiology, 86(6), 2013–2018. 10.1152/jappl.1999.86.6.2013 10368368

[R19] Hill JM, Pickar JG, Parrish MD, & Kaufman MP (1992). Effects of hypoxia on the discharge of group III and IV muscle afferents in cats. Journal of Applied Physiology, 73(6), 2524–2529. 10.1152/jappl.1992.73.6.2524 1490966

[R20] Hogan MC, Arthur PG, Bebout DE, Hochachka PW, & Wagner PD (1992). Role of O2 in regulating tissue respiration in dog muscle working in situ. Journal of Applied Physiology, 73(2), 728–736. 10.1152/jappl.1992.73.2.728 1400003

[R21] Hogan MC, Richardson RS, & Haseler LJ (1999). Human muscle performance and PCr hydrolysis with varied inspired oxygen fractions: A 31P-MRS study. Journal of Applied Physiology, 86(4), 1367–1373. 10.1152/jappl.1999.86.4.1367 10194224

[R22] Hureau TJ, Weavil JC, Thurston TS, Broxterman RM, Nelson AD, Bledsoe AD, Jessop JE, Richardson RS, Wray DW, & Amann M (2018). Identifying the role of group III/IV muscle afferents in the carotid baroreflex control of mean arterial pressure and heart rate during exercise. The Journal of Physiology, 596(8), 1373–1384. 10.1113/JP275465 PMC5899981 29388218

[R23] Hureau TJ, Weavil JC, Thurston TS, Wan H-Y, Gifford JR, Jessop JE, Buys MJ, Richardson RS, & Amann M (2019). Pharmacological attenuation of group III/IV muscle afferents improves endurance performance when oxygen delivery to locomotor muscles is preserved. Journal of Applied Physiology, 127(5), 1257–1266. 10.1152/japplphysiol.00490.2019 PMC6879838 31513446

[R24] Iannetta D, Weavil JC, Laginestra FG, Thurston TS, Broxterman RM, Jenkinson RH, Curtis MC, Chang J, Wan HY, & Amann M (2024). Control of hyperpnoea and pulmonary gas exchange during prolonged exercise: The role of group III/IV muscle afferent feedback. The Journal of Physiology, 602(20), 5375–5389. 10.1113/JP286993 PMC11493515 39316014

[R25] Jankowski MP, Rau KK, Ekmann KM, Anderson CE, & Koerber HR (2013). Comprehensive phenotyping of group III and IV muscle afferents in mouse. Journal of Neurophysiology, 109(9), 2374–2381. 10.1152/jn.01067.2012 PMC3652219 23427306

[R26] Juel C, Lundby C, Sander M, Calbet J, & van Hall G (2003). Human skeletal muscle and erythrocyte proteins involved in acid-base homeostasis: Adaptations to chronic hypoxia. The Journal of Physiology, 548(2), 639–648. 10.1113/jphysiol.2002.035899 PMC2342856 12611920

[R27] Katz A, & Sahlin K (1987). Effect of decreased oxygen availability on NADH and lactate contents in human skeletal muscle during exercise. Acta Physiologica Scandinavica, 131(1), 119–127. 10.1111/j.1748-1716.1987.tb08213.x 3673605

[R28] Krapf R, Beeler I, Hertner D, & Hulter HN (1991). Chronic respiratory alkalosis: The effect of sustained hyperventilation on renal regulation of acid–base equilibrium. New England Journal of Medicine, 324(20), 1394–1401. 10.1056/NEJM199105163242003 1902283

[R29] Lalley PM (2008). Opioidergic and dopaminergic modulation of respiration. Respiratory Physiology & Neurobiology, 164(1–2), 160–167. 10.1016/j.resp.2008.02.004 PMC2642894 18394974

[R30] Light AR, Hughen RW, Zhang J, Rainier J, Liu Z, & Lee J (2008). Dorsal root ganglion neurons innervating skeletal muscle respond to physiological combinations of protons, ATP, and lactate mediated by ASIC, P2X, and TRPV1. Journal of Neurophysiology, 100(3), 1184–1201. 10.1152/jn.01344.2007 PMC6195653 18509077

[R31] López-Barneo J, González-Rodríguez P, Gao L, Fernández-Agüera M, Pardal R, & Ortega-Sáenz P (2016). Oxygen sensing by the carotid body: Mechanisms and role in adaptation to hypoxia. American Journal of Physiology Cell Physiology, 310(8), C629–C642. 10.1152/ajpcell.00265.2015 26764048

[R32] López-Barneo J, Ortega-Sáenz P, Pardal R, Pascual A, & Piruat J (2008). Carotid body oxygen sensing. European Respiratory Journal, 32(5), 1386–1398. 10.1183/09031936.00056408 18978138

[R33] Lundby C, Calbet JA, & Robach P (2009). The response of human skeletal muscle tissue to hypoxia. Cellular and Molecular Life Sciences, 66(22), 3615–3623. 10.1007/s00018-009-0146-8 PMC11115669 19756383

[R34] Lundby C, Calbet JAL, van Hall G, Saltin B, & Sander M (2004). Pulmonary gas exchange at maximal exercise in Danish lowlanders during 8 wk of acclimatization to 4,100 m and in high-altitude Aymara natives. American Journal of Physiology-Regulatory, Integrative and Comparative Physiology, 287(5), R1202–R1208. 10.1152/ajpregu.00725.2003 15191909

[R35] Mizuno M, Savard GK, Areskog N-H, Lundby C, & Saltin B (2008). Skeletal muscle adaptations to prolonged exposure to extreme altitude: A role of physical activity? High Altitude Medicine & Biology, 9(4), 311–317. 10.1089/ham.2008.1009 19115916

[R36] Parolin M, Spriet L, Hultman E, Hollidge-Horvat M, Jones N, & Heigenhauser G (2000). Regulation of glycogen phosphorylase and PDH during exercise in human skeletal muscle during hypoxia. American Journal of Physiology Endocrinology and Metabolism, 278(3), E522–E534. 10.1152/ajpendo.2000.278.3.E522 10710508

[R37] Roche J, Rasmussen P, Gatterer H, Roveri G, Turner R, Hall G. v., Maillard M, Walzl A, Kob M, Strapazzon G, Goetze JP, Schäfer ST, Kammerer T, Nader E, Connes P, Robert M, Mueller T, Feraille E, & Siebenmann C (2022). Hypoxia briefly increases diuresis but reduces plasma volume by fluid redistribution in women. American Journal of Physiology-Heart and Circulatory Physiology, 323(6), 1068–1079. 10.1152/ajpheart.00394.2022 PMC9678412 36269645

[R38] Schlittler M, Gatterer H, Turner R, Regli IB, Woyke S, Strapazzon G, Rasmussen P, Kob M, Mueller T, Goetze JP, Maillard M, van Hall G, Feraille E, & Siebenmann C (2021). Regulation of plasma volume in male lowlanders during 4 days of exposure to hypobaric hypoxia equivalent to 3500 m altitude. The Journal of Physiology, 599(4), 1083–1096. 10.1113/JP280601 PMC7894546 33124686

[R39] Severinghaus JW (1966). Blood gas calculator. Journal of Applied Physiology, 21(3), 1108–1116. 10.1152/jappl.1966.21.3.1108 5912737

[R40] Siebenmann C, Cathomen A, Hug M, Keiser S, Lundby A, Hilty M, Goetze J, Rasmussen P, & Lundby C (2015). Hemoglobin mass and intravascular volume kinetics during and after exposure to 3,454-m altitude. Journal of Applied Physiology, 119(10), 1194–1201. 10.1152/japplphysiol.01121.2014 25749449

[R41] Siebenmann C, Roche J, Schlittler M, Simpson LL, & Stembridge M (2024). Regulation of haemoglobin concentration at high altitude. The Journal of Physiology, 602(21), 5587–5600. 10.1113/JP284578 38051656

[R42] Stickney JC, & Van Liere EJ (1953). Acclimatization to low oxygen tension. Physiological Reviews, 33(1), 13–34. 10.1152/physrev.1953.33.1.13 13013831

[R43] Thurston TS, Weavil JC, Georgescu VP, Wan H-Y, Birgenheier NM, Morrissey CK, Jessop JE, & Amann M (2023). The exercise pressor reflex – a pressure-raising mechanism with a limited role in regulating leg perfusion during locomotion in young healthy men. The Journal of Physiology, 601(20), 4557–4572. 10.1113/JP284870 PMC10592099 37698303

[R44] van Hall G, Lundby C, Araoz M, Calbet JA, Sander M, & Saltin B (2009). The lactate paradox revisited in lowlanders during acclimatization to 4100 m and in high-altitude natives. The Journal of Physiology, 587(Pt 5), 1117–1129. 10.1113/jphysiol.2008.160846 PMC2673779 19139048

[R45] Vízek M, Pickett C, & Weil J (1987). Increased carotid body hypoxic sensitivity during acclimatization to hypobaric hypoxia. Journal of Applied Physiology, 63(6), 2403–2410. 10.1152/jappl.1987.63.6.2403 3436874

[R46] Wan H-Y, Weavil JC, Thurston TS, Georgescu VP, Bledsoe AD, Jessop JE, Buys MJ, Richardson RS, & Amann M (2020a). The muscle reflex and chemoreflex interaction: Ventilatory implications for the exercising human. Journal of Applied Physiology, 129(4), 691–700. 10.1152/japplphysiol.00449.2020 PMC7654695 32816637

[R47] Wan HY, Weavil JC, Thurston TS, Georgescu VP, Hureau TJ, Bledsoe AD, Buys MJ, Jessop JE, Richardson RS, & Amann M (2020b). The exercise pressor reflex and chemoreflex interaction: Cardiovascular implications for the exercising human. The Journal of Physiology, 598(12), 2311–2321. 10.1113/JP279456 PMC7720421 32170732

