# Supplementary material for: The role of group III/IV afferent feedback in the ventilatory, pressor and metabolic responses to exercise in normoxia and hypoxia
Source: J Physiol. Author manuscript; Available in PMC 2026 Sep 19. (PMC13589313; doi:10.1113/JP290914)
Supplement: Supplementary file 1 [file NIHMS2205800-supplement-Supplementary_material.pdf]

The Journal of Physiology

The role of group III/IV afferent feedback in the ventilatory, pressor, and metabolic responses to exercise in normoxia and hypoxia

Elliott J Jenkins, Phuong L Ha, Connor A Howe, Jay M.J.R. Carr, Justin A Monteleone, Andrew JM Douglas, Abigail Nicholson, Andrew R Steele, Brydie Lemkes, Jodie Lauren Koep, L. Madden Brewster, Jennifer Duffy, Mathew Ian Bergens Debenham, Tenasia Monaghan, Declan J H Isaak, Jacob McCormick, Brian H Dalton, Chris J McNeil, Jared Baylis, David B MacLeod, Mike Stembridge, Markus Amann, Philip N Ainslie, and Travis D Gibbons
DOI: 10.1113/JP290914

Corresponding author(s): Elliott Jenkins (ejenkins2@cardiffmet.ac.uk)

The referees have opted to remain anonymous.

Review Timeline:

Submission Date:	12-Jan-2026
Editorial Decision:	26-Feb-2026
Revision Received:	15-Apr-2026
Editorial Decision:	05-May-2026
Revision Received:	07-May-2026
Accepted:	15-May-2026

Senior Editor: Kim Barrett

Reviewing Editor: Ken O'Halloran

Transaction Report:

(Note: With the exception of the correction of typographical or spelling errors that could be a source of ambiguity, letters and reports are not edited. Depending on transfer agreements, referee reports obtained elsewhere may or may not be included in this compilation. Referee reports are anonymous unless the Referee chooses to sign their reports.)

Dear Dr Jenkins,

Re: JP-RP-2026-290914 "The role of group III/IV afferent feedback in the ventilatory, pressor, and metabolic responses to exercise in normoxia and hypoxia" by Elliott J Jenkins, Phuong L Ha, Connor A Howe, Jay M.J.R. Carr, Justin A Monteleone, Andrew JM Douglas, Abigail Nicholson, Andrew R Steele, Brydie Lemkes, Jodie Lauren Koep, L. Madden Brewster, Jennifer Duffy, Mathew Ian Bergens Debenham, Tenasia Monaghan, Declan Isaak, Jacob McCormick, Brian H Dalton, Chris J McNeil, Jared Baylis, David B MacLeod, Mike Stembridge, Philip N Ainslie, Markus Amann, and Travis D Gibbons

Thank you for submitting your manuscript to The Journal of Physiology. It has been assessed by a Reviewing Editor and by 2 expert referees and we are pleased to tell you that it is acceptable for publication following satisfactory revision.

The referee reports are copied at the end of this email.

Please address all the points raised and incorporate all requested revisions or explain in your Response to Referees why a change has not been made. We hope you will find the comments helpful and that you will be able to return your revised manuscript within 4 weeks. If you require longer than this, please contact journal staff: jp@physoc.org.

Your revised manuscript should be submitted online using the link in your Author Tasks Link Not Available. This link is accessible via your account as Corresponding Author; it is not available to your co-authors. If this presents a problem, please contact journal staff (jp@physoc.org). Image files from the previous version are retained on the system. Please ensure you replace or remove any files that are being revised.

If you do not wish to submit a revised version of your manuscript, you must inform our journal staff (jp@physoc.org) or reply to this email to request withdrawal. Please note that a manuscript must be formally withdrawn from the peer review process at one journal before it may be submitted to another journal.

TRANSPARENT PEER REVIEW POLICY: To improve the transparency of its peer review process The Journal of Physiology publishes online, as supporting information, the peer review history of all articles accepted for publication. Readers will have access to decision letters, including Editors' comments and referee reports, for each version of the manuscript, as well as any author responses to peer review comments. Referees can decide whether or not they wish to be named on the peer review history document.

ABSTRACT FIGURES: Authors are expected to use The Journal's premium BioRender account to create/redraw their Abstract Figures. Information on how to access this account is here: https://physoc.onlinelibrary.wiley.com/journal/14697793/biorender-access.

This will enable Authors to create and download high-resolution figures. If authors have used the free BioRender service, they can use the instructions provided in the link above to download a high-resolution version suitable for publication.

The link provided should only be used for the purposes of this submission. Authors will be charged for figures created on this account if they are not related to this manuscript submission.

LANGUAGE EDITING AND SUPPORT FOR PUBLICATION: If you would like help with English language editing, or other article preparation support, Wiley Editing Services offers expert help, including English Language Editing, as well as translation, manuscript formatting, and figure formatting at www.wileyauthors.com/eeo/preparation. You can also find resources for Preparing Your Article for general guidance about writing and preparing your manuscript at www.wileyauthors.com/eeo/prepresources.

RIGOUR AND REPRODUCIBILITY: We recommend authors consult The Journal's Rigour and Reproducibility requirements to ensure that their manuscript is suitable for publication: https://physoc.onlinelibrary.wiley.com/pb-assets/hub-assets/physoc/documents/TJP-Rigour-and-Reproducibility-Requirements.pdf

REVISION CHECKLIST:

Check that your Methods section conforms to journal policy: https://jp.msubmit.net/cgi-bin/main.plex?form_type=display_requirements#methods

Check that data presented conforms to the statistics policy: https://jp.msubmit.net/cgi-bin/main.plex?form_type=display_requirements#statistics

Upload a full Response to Referees file. To create your 'Response to Referees' copy all the reports, including any comments from the Senior and Reviewing Editors, into a Microsoft Word, or similar, file and respond to each point, using font or background colour to distinguish comments and responses and upload as the required file type.

Please upload two versions of your manuscript text: one with all relevant changes highlighted and one clean version with no changes tracked. The manuscript file should include all tables and figure legends, but each figure/graph should be uploaded

as separate, high-resolution files. The journal is now integrated with Wiley's Image Checking service. For further details, see: https://www.wiley.com/en-us/network/publishing/research-publishing/trending-stories/upholding-image-integrity-wileys-image-screening-service

You may also upload:

- 'Potential Cover Art' for consideration as the issue's cover image
- Appropriate Supporting Information (Video, audio or data set: see https://jp.msubmit.net/cgi-bin/main.plex?form_type=display_requirements#supp).

We look forward to receiving your revised submission.

If you have any queries, please reply to this email and we will be pleased to advise.

Yours sincerely,

Kim Barrett
Senior Editor
The Journal of Physiology

REQUIRED ITEMS

- Author photo and profile. First or joint first authors are asked to provide a short biography (no more than 100 words for one author or 150 words in total for joint first authors) and a portrait photograph. These should be uploaded and clearly labelled together in a Word document with the revised version of the manuscript. See Information for Authors for further details.

- Please upload separate high-quality figure files via the submission form.

- Please include an Abstract Figure file and an Abstract Figure legend. An appropriate figure legend, which should not exceed 150 words in length, should be included in the main manuscript file. The Abstract Figure is a piece of artwork designed to give readers an immediate understanding of the research and should summarise the main conclusions. If possible, the image should be easily 'readable' from left to right or top to bottom. It should show the physiological relevance of the manuscript so readers can assess the importance and content of its findings. Abstract Figures should not merely recapitulate other figures in the manuscript. Please try to keep the diagram as simple as possible and without superfluous information that may distract from the main conclusion(s). Abstract Figures must be provided by authors no later than the revised manuscript stage and should be uploaded as a separate file during online submission labelled as File Type 'Abstract Figure'. Please also ensure that you include the figure legend in the main article file. All Abstract Figures should be created using BioRender. Authors should use The Journal's premium BioRender account to export high-resolution images. Details on how to use and access the premium account are included as part of this email.

EDITOR COMMENTS

Reviewing Editor:

Thank you for submitting your manuscript to The Journal of Physiology. I am pleased to report that the paper was generally well received by expert referees who recognise the technically challenging aspect of the work. Both referees commend the authors on the execution of a fine study. Fair-spirited, constructive critiques are provided. Notwithstanding the evidence portrayed in the manuscript, R1 highlights that the finding of a near blunting of exercise pressor response following group III/IV afferent blockade is surprising given the broader integrative framework for BP control. Therefore, the referee challenges the authors to resolve this apparent contradiction and/or provide for a more inclusive approach acknowledging other contributory factors (base don existing literature). You may choose, of course, to provide a robust rebuttal and strongly defend the existing claims and conclusions based on the data in the manuscript. R1 highlights concern with the lack of cardiovascular assessment concurrent with the assessment of potential cephalic spread of fentanyl. It is strongly suggested that this limitation might be expected to temper the strength of the authors' conviction of the strength of their data.

R2 raises important points that should also be carefully addressed.

I look forward to receiving your responses, which should be point-by-point to the issues raised, and a revised manuscript.

Please also see 'Required Items' above.

REFEREE COMMENTS

Referee #1:

The study addresses an important and timely question regarding the role of group III/IV muscle afferent feedback in ventilatory and cardiovascular regulation during exercise under normoxic and hypoxic conditions, including the influence of acclimatisation. The combined use of hypoxia, acclimatisation, and intrathecal fentanyl represents a technically demanding and potentially impactful approach within the field of integrative exercise physiology. The experimental design is carefully executed, the dataset is extensive and internally consistent, and the ventilatory findings provide meaningful extensions to the existing literature.

However, the overall impact of the study is limited by interpretative issues related to the cardiovascular findings, particularly the pressor response. While the manuscript offers descriptive insight into ventilatory control, the mechanistic interpretation of blood pressure regulation is problematic. Blood pressure during exercise reflects the integrated outcome of central command, baroreflex resetting, vascular transduction, and baseline hemodynamic state. In this context, the near-complete abolition of the pressor response following intrathecal fentanyl, despite preserved heart rate responses and across multiple exercise intensities, oxygen conditions, and acclimatisation states, is physiologically difficult to reconcile with partial attenuation of group III/IV muscle afferent feedback alone.

Prior human studies consistently demonstrate that attenuation of group III/IV afferent input reduces, but does not abolish, the pressor response to exercise, even at low intensities. The manuscript does not adequately reconcile these findings with the near-complete suppression of the pressor response observed here, nor does it sufficiently consider additional mechanisms that may contribute to the observed hemodynamic responses.

Although the originality of combining intrathecal fentanyl with hypoxic exercise and acclimatisation is a strength, the lack of blood pressure measurements during the specific assessment of potential cephalad spread of fentanyl further limits the robustness of the cardiovascular interpretation. Cardiovascular variables were not assessed during this control experiment, which restricts the ability to exclude subtle central effects on vasomotor control and sympathetic regulation. Further, the use of a >2 SD criterion for ventilatory responses appears to represent a statistical threshold rather than a physiologically validated benchmark. While this approach may be appropriate for identifying atypical changes in ventilation, it may be less suited for excluding potential central cardiovascular effects of intrathecal fentanyl, given that ventilatory and vasomotor control are not necessarily tightly coupled.

Taken together, while the experimental data are robust and the ventilatory conclusions are generally supported, the interpretation of the cardiovascular findings raises a fundamental conceptual concern that substantially limits the validity of the conclusions regarding cardiovascular regulation. In its present form, the mechanistic interpretation requires careful reconsideration, and a more explicit and integrative discussion of alternative mechanisms consistent with closed-loop blood pressure regulation would be necessary to adequately support the conclusions.

Referee #2:

The study by Jenkins and colleagues investigates the contribution of group III/IV skeletal muscle afferents to the cardiovascular, respiratory and metabolic responses to leg cycling exercise in acute (PIO2=90 mmHg) and chronic (7-9 days at 3,600m, PIO2=90 mmHg) hypoxia. These are complex and demanding experiments undertaken by researchers with a strong track record in undertaken such invasive human studies. The line of enquiry followed builds authentically upon previous work by the team investigating the functional significance of group III/IV skeletal muscle afferents using intrathecal fentanyl (e.g., Amman) and the physiological responses to conditions of low oxygen (e.g., Ainslie and many others). The manuscript is well written, and care has been taken to clearly communicate this rich data set. I have only relatively minor comments:

-The experimental design is very complex involving three exercise intensities (~90W, ~135W, ~185W), two conditions (saline, fentanyl), and three trials (normoxia, sea level hypoxia, high altitude hypoxia). Please can the authors clarify the rationale for including four fixed effects (i.e., intensity, condition, PIO2, acclimatization) in the linear mixed-effects model? Specifically, the practice of comparing normoxia, sea level hypoxia, high altitude hypoxia in terms of both PIO2 and acclimatization fixed-effects. In addition, while it is recognised that conducting such studies on n=14 individuals is a considerable undertaking, it should be considered whether more subtle interactions might have been missed due to a lack of statistical power. Also, why where "independent t-tests" used to examine the effects of fentanyl - as the same individuals are studied in both conditions presumably dependent (paired-samples) t-test would be appropriate.

-Introduction lines 114-125, do not seem necessary. The introduction is already quite long, patients are not being studied, and the argument being made is arguably tangential. Given how well the preceding section is set up this is distracting and it would probably be preferable to omit and lead straight into the aims/hypotheses paragraph.

-Why was a hyperoxia trial performed at high altitude (line 217), but the data are not included? (If included, I could understand better why four fixed-factors were included).

-Results. It is striking how carefully SaO2 (%) is maintained at rest for sea level and high-altitude trials and yet exercise under both hypoxic conditions yields remarkable individual differences in the responses. Given the authors conclusions (e.g., 303-305), is this inter-individual variability related to variations in the fentanyl-induced suppression of ventilation?

-Figure 7. Please could the authors comment on the differential effects of fentanyl on lactate and [H+]. It is intriguing that while lactate is only increased by fentanyl in acute hypoxia, [H+] is increased in all three scenarios.

-Other candidates for including in the "Methodological considerations" section. 1) Three participants were unavailable for sea level testing. 2) Three months separate sea level and high-altitude conditions. 3) Statistical power [as per comment above]. 4) Potential carry over effects of not randomising/counter balancing conditions. 5) Potential contribution of the elevated "pre-exercise" physiological state on the exercise responses reported [SBP is extremely high].

Minor typographical suggestions:

-Given the complexity of the statistical design, it would be helpful if when P values are provided in the text (e.g., abstract, results) the specific interaction referred to is stated. This is often the practice but not applied universally. Also, it would be helpful if the term "condition" were defined more clearly and used more consistently when referring to control/fentanyl, given that there are multiple potential conditions to which the authors could be referring (very minor but will help avoid ambiguity).

-As hypotension technically refers to a specific clinical state, to avoid confusion it would be helpful to reword statements such as "greater hypotensive effect" etc as "greater blood pressure lowering effect" (or similar).

-The phrasing around the rest period was a little ambiguous. In the results section (line 296) "true resting conditions (i.e., prior to the onset of cycling)" are mentioned, which presumably is the "resting baseline" mentioned on line 185, rather than

baselines described on lines 197-204. Both baselines are conducted before cycling, and using the term "resting baseline" consistently may avoid ambiguity.

-Please state specific packages used with R for statistical analysis.

END OF COMMENTS

Dear Dr Jenkins,

Re: JP-RP-2026-290914 "The role of group III/IV afferent feedback in the ventilatory, pressor, and metabolic responses to exercise in normoxia and hypoxia" by Elliott J Jenkins, Phuong L Ha, Connor A Howe, Jay M.J.R. Carr, Justin A Monteleone, Andrew JM Douglas, Abigail Nicholson, Andrew R Steele, Brydie Lemkes, Jodie Lauren Koep, L. Madden Brewster, Jennifer Duffy, Mathew Ian Bergens Debenham, Tenasia Monaghan, Declan Isaak, Jacob McCormick, Brian H Dalton, Chris J McNeil, Jared Baylis, David B MacLeod, Mike Stembridge, Philip N Ainslie, Markus Amann, and Travis D Gibbons

Thank you for submitting your manuscript to The Journal of Physiology. It has been assessed by a Reviewing Editor and by 2 expert referees and we are pleased to tell you that it is acceptable for publication following satisfactory revision.

The referee reports are copied at the end of this email.

Please address all the points raised and incorporate all requested revisions or explain in your Response to Referees why a change has not been made. We hope you will find the comments helpful and that you will be able to return your revised manuscript within 4 weeks. If you require longer than this, please contact journal staff: jp@physoc.org.

Your revised manuscript should be submitted online using the link in your Author Tasks https://jp.msubmit.net/cgi-bin/main.plex?el=A3JS3Hwq1A1baa4F3A9ftdbIadOPxAmSylpFV7ffKdDAZ. This link is accessible via your account as Corresponding Author; it is not available to your co-authors. If this presents a problem, please contact journal staff (jp@physoc.org). Image files from the previous version are retained on the system. Please ensure you replace or remove any files that are being revised.

If you do not wish to submit a revised version of your manuscript, you must inform our journal staff (jp@physoc.org) or reply to this email to request withdrawal. Please note that a manuscript must be formally withdrawn from the peer review process at one journal before it may be submitted to another journal.

TRANSPARENT PEER REVIEW POLICY: To improve the transparency of its peer review process The Journal of Physiology publishes online, as supporting information, the peer review history of all articles accepted for publication. Readers will have access to decision letters, including Editors' comments and referee reports, for each version of the manuscript, as well as any author responses to peer review comments. Referees can decide whether or not they wish to be named on the peer review history document.

ABSTRACT FIGURES: Authors are expected to use The Journal's premium BioRender account to create/redraw their Abstract Figures. Information on how to access this account is here: https://physoc.onlinelibrary.wiley.com/journal/14697793/biorender-access.

This will enable Authors to create and download high-resolution figures. If authors have used the free BioRender service, they can use the instructions provided in the link above to

download a high-resolution version suitable for publication.

The link provided should only be used for the purposes of this submission. Authors will be charged for figures created on this account if they are not related to this manuscript submission.

LANGUAGE EDITING AND SUPPORT FOR PUBLICATION: If you would like help with English language editing, or other article preparation support, Wiley Editing Services offers expert help, including English Language Editing, as well as translation, manuscript formatting, and figure formatting at www.wileyauthors.com/eeo/preparation. You can also find resources for Preparing Your Article for general guidance about writing and preparing your manuscript at www.wileyauthors.com/eeo/prepresources.

RIGOUR AND REPRODUCIBILITY: We recommend authors consult The Journal's Rigour and Reproducibility requirements to ensure that their manuscript is suitable for publication: https://physoc.onlinelibrary.wiley.com/pb-assets/hub-assets/physoc/documents/TJP-Rigour-and-Reproducibility-Requirements.pdf

REVISION CHECKLIST:

Check that your Methods section conforms to journal policy: https://jp.msubmit.net/cgi-bin/main.plex?form_type=display_requirements#methods

Check that data presented conforms to the statistics policy: https://jp.msubmit.net/cgi-bin/main.plex?form_type=display_requirements#statistics

Upload a full Response to Referees file. To create your 'Response to Referees' copy all the reports, including any comments from the Senior and Reviewing Editors, into a Microsoft Word, or similar, file and respond to each point, using font or background colour to distinguish comments and responses and upload as the required file type.

Please upload two versions of your manuscript text: one with all relevant changes highlighted and one clean version with no changes tracked. The manuscript file should include all tables and figure legends, but each figure/graph should be uploaded as separate, high-resolution files. The journal is now integrated with Wiley's Image Checking service. For further details, see: https://www.wiley.com/en-us/network/publishing/research-publishing/trending-stories/upholding-image-integrity-wileys-image-screening-service

You may also upload:

- 'Potential Cover Art' for consideration as the issue's cover image
- Appropriate Supporting Information (Video, audio or data set: see https://jp.msubmit.net/cgi-bin/main.plex?form_type=display_requirements#supp).

We look forward to receiving your revised submission.

If you have any queries, please reply to this email and we will be pleased to advise.

Yours sincerely,

Kim Barrett
Senior Editor
The Journal of Physiology

REQUIRED ITEMS

- Author photo and profile. First or joint first authors are asked to provide a short biography (no more than 100 words for one author or 150 words in total for joint first authors) and a portrait photograph. These should be uploaded and clearly labelled together in a Word document with the revised version of the manuscript. See Information for Authors for further details.

A joint author bio and portrait photographs have been added to the revised version of the manuscript.

- Please upload separate high-quality figure files via the submission form.

Separate high quality figures have been uploaded.

- Please include an Abstract Figure file and an Abstract Figure legend. An appropriate figure legend, which should not exceed 150 words in length, should be included in the main manuscript file. The Abstract Figure is a piece of artwork designed to give readers an immediate understanding of the research and should summarise the main conclusions. If possible, the image should be easily 'readable' from left to right or top to bottom. It should show the physiological relevance of the manuscript so readers can assess the importance and content of its findings. Abstract Figures should not merely recapitulate other figures in the manuscript. Please try to keep the diagram as simple as possible and without superfluous information that may distract from the main conclusion(s). Abstract Figures must be provided by authors no later than the revised manuscript stage and should be uploaded as a separate file during online submission labelled as File Type 'Abstract Figure'. Please also ensure that you include the figure legend in the main article file. All Abstract Figures should be created using BioRender. Authors should use The Journal's premium BioRender account to export high-resolution images. Details on how to use and access the premium account are included as part of this email.

An abstract figure, and accompanying figure legend have been included in the revised submission.

EDITOR COMMENTS

Reviewing Editor:

Thank you for submitting your manuscript to The Journal of Physiology. I am pleased to report that the paper was generally well received by expert referees who recognise the technically challenging aspect of the work. Both referees commend the authors on the execution of a fine study. Fair-spirited, constructive critiques are provided. Notwithstanding the evidence portrayed in the manuscript, R1 highlights that the finding of a near blunting of exercise pressor response following group III/IV afferent blockade is surprising given the broader integrative framework for BP control. Therefore, the referee challenges the authors to resolve this apparent contradiction and/or provide for a more inclusive approach acknowledging other contributory factors (based on existing literature). You may choose, of course, to provide a robust rebuttal and strongly defend the existing claims and conclusions based on the data in the manuscript. R1 highlights concern with the lack of cardiovascular assessment concurrent with the assessment of potential cephalic spread of fentanyl. It is strongly suggested that this limitation might be expected to temper the strength of the authors' conviction of the strength of their data.

We thank the Reviewing Editor for the positive evaluation and for highlighting the concerns raised by Referee 1.

In response, we have revised the Discussion to provide a more integrative interpretation of the cardiovascular findings, including alternative mechanisms that contribute to exercising blood pressure and heart rate responses.

At the same time, we stand by the magnitude and consistency of the observed blood pressure response, which was robust across participants and particularly pronounced in hypoxia. We highlight that the use of large muscle mass rhythmic exercise under systemic hypoxia, together with direct brachial arterial pressure measurement, likely contributes to the larger effect relative to prior studies.

With respect to the concern regarding potential cephalad migration of fentanyl, we have incorporated additional cardiovascular data from the arm-crank control experiment (HR), which showed no effect of fentanyl, and note that direct brachial artery blood pressure was measured but not interpretable due to movement artefact during arm ergometry. Additionally, the absence of any effect of fentanyl at rest on blood pressure or heart rate further supports the absence of a central cardiovascular influence of intrathecal fentanyl.

We hope these revisions adequately address the concerns raised while preserving the key findings of the study.

R2 raises important points that should also be carefully addressed.

We thank the Reviewing Editor for highlighting the constructive comments from Referee 2.

All points raised have been carefully addressed. We have clarified the statistical modelling approach and terminology, revised sections of the Introduction to improve focus, and

expanded the Methodological considerations to address limitations related to study design and statistical power. We have also clarified the rationale for excluding the hyperoxia condition and provided additional interpretation of the lactate and [H^+] responses.

Minor revisions have been made throughout to improve clarity and consistency, including more explicit reporting of interaction terms, consistent use of terminology (e.g., “condition”, “resting baseline”), and specification of the statistical packages used.

We believe these revisions have improved the clarity and transparency of the manuscript.

I look forward to receiving your responses, which should be point-by-point to the issues raised, and a revised manuscript.

Please also see 'Required Items' above.

REFEREE COMMENTS

Referee #1:

The study addresses an important and timely question regarding the role of group III/IV muscle afferent feedback in ventilatory and cardiovascular regulation during exercise under normoxic and hypoxic conditions, including the influence of acclimatisation. The combined use of hypoxia, acclimatisation, and intrathecal fentanyl represents a technically demanding and potentially impactful approach within the field of integrative exercise physiology. The experimental design is carefully executed, the dataset is extensive and internally consistent, and the ventilatory findings provide meaningful extensions to the existing literature.

We thank the referee for their positive assessment of the study and for recognising its contribution to the literature.

However, the overall impact of the study is limited by interpretative issues related to the cardiovascular findings, particularly the pressor response. While the manuscript offers descriptive insight into ventilatory control, the mechanistic interpretation of blood pressure regulation is problematic. Blood pressure during exercise reflects the integrated outcome of central command, baroreflex resetting, vascular transduction, and baseline hemodynamic state. In this context, the near-complete abolition of the pressor response following intrathecal fentanyl, despite preserved heart rate responses and across multiple exercise intensities, oxygen conditions, and acclimatisation states, is physiologically difficult to reconcile with partial attenuation of group III/IV muscle afferent feedback alone.

We thank the reviewer for this thoughtful comment. We agree that arterial pressure during exercise reflects the integrated output of multiple control systems, and we have revised the Discussion to better reflect this. Specifically, we now frame the reduction in MAP with intrathecal fentanyl as the net haemodynamic consequence of attenuated afferent input, rather than attributing the response to a single regulatory pathway.

Importantly, we would like to emphasise that we stand by the magnitude of the blood pressure response observed in the present study. The inhibitory effect of intrathecal fentanyl was consistent across participants and experimental conditions, and was particularly pronounced in hypoxia, which represents a novel finding. Regarding the latter, our interpretation is that group III/IV afferent feedback plays an integral role in counteracting local hypoxic vasodilation via its role in the pressor response.

There are several methodological and physiological factors that may explain the larger reduction in MAP compared to previous work. First, arterial pressure was measured via a brachial arterial catheter, which provides a more direct estimate of central pressure vs radial measurements commonly used in prior studies (e.g., Hureau et al. (2018)). Second, our model employed large muscle mass upright cycling under systemic hypoxia, which likely imposes a greater global haemodynamic and sympathetic demand when compared to small muscle mass exercise models used in prior work. Under these conditions, the overall contribution of group III/IV afferent feedback to the maintenance of exercising blood pressure will be amplified.

Taken together, we believe that the large magnitude of the inhibitory effect of intrathecal fentanyl on the pressor response reflects the specific physiological context of the present study, rather than a discrepancy in the underlying mechanisms or cephalad migration.

Prior human studies consistently demonstrate that attenuation of group III/IV afferent input reduces, but does not abolish, the pressor response to exercise, even at low intensities. The manuscript does not adequately reconcile these findings with the near-complete suppression of the pressor response observed here, nor does it sufficiently consider additional mechanisms that may contribute to the observed hemodynamic responses.

We agree with the reviewer that prior studies generally report a reduction, rather than abolition, of the pressor response following attenuation of group III/IV afferent feedback.

As mentioned above, we note that differences in experimental design may contribute to the magnitude of the observed effect. Previous studies (e.g., Hureau et al. (2018)) have largely employed electrically evoked or small muscle mass exercise, where MAP typically increases modestly. In contrast, the present study used large muscle mass dynamic exercise, where the maintenance of arterial pressure depends more heavily on systemic vascular control. Under these conditions, attenuation of afferent-mediated sympathetic vasoconstriction may result in a proportionally larger reduction in MAP. We are not aware of previous studies examining the effect of intrathecal fentanyl on arterial pressure during whole-body exercise in hypoxia, and therefore direct comparisons with prior normoxic or small muscle mass models should be made with caution.

Additionally, the exaggerated depressor response observed in hypoxia suggests that afferent feedback plays a more prominent role in supporting arterial pressure when oxygen availability is reduced, which may not be captured in normoxic models.

Finally, prior work (Amann et al., 2010) proposed that the influence of group III/IV afferents would be greatest at high exercise intensities, where the metabolic stimulus is maximal. However, this was not observed, and it was suggested that alternative mechanisms contribute more substantially to both ventilatory and pressor responses as exercise intensity increases. To reconcile these findings with the present data, it is important to consider the relative exercise intensities employed. The workloads used in the current study were substantially lower than those in previous work (e.g., ~325 W), and therefore may not have recruited these alternative mechanisms to the same extent. Under these conditions, the relative contribution of afferent feedback may be greater, allowing a more pronounced and progressive effect of intrathecal fentanyl on MAP to be observed with increasing exercise intensity.

Although the originality of combining intrathecal fentanyl with hypoxic exercise and acclimatisation is a strength, the lack of blood pressure measurements during the specific assessment of potential cephalad spread of fentanyl further limits the robustness of the cardiovascular interpretation. Cardiovascular variables were not assessed during this control experiment, which restricts the ability to exclude subtle central effects on vasomotor control and sympathetic regulation. Further, the use of a >2 SD criterion for ventilatory responses appears to represent a statistical threshold rather than a physiologically validated benchmark. While this approach may be appropriate for identifying atypical changes in ventilation, it may be less suited for excluding potential central cardiovascular effects of intrathecal fentanyl, given that ventilatory and vasomotor control are not necessarily tightly coupled.

We thank the referee for this important comment. We agree that the assessment of potential cephalad spread of intrathecal fentanyl is critical for interpreting the cardiovascular findings.

During the arm-crank control experiment, cardiovascular variables were assessed. Heart rate responses were not different between fentanyl and control conditions (118 vs 121 bpm, p = 0.148), providing further support that intrathecal fentanyl did not exert meaningful central effects on cardiovascular regulation. These data have now been incorporated into the Methods and Results.

Arterial blood pressure was also measured during this control experiment; however, due to the positioning of the arterial catheter and the nature of arm-crank exercise, a majority of these signals were highly variable (large movement artefact) and did not provide a reliable estimate of arterial pressure. We therefore elected not to include these data in the manuscript and have clarified this in the Methods.

Additionally, intrathecal fentanyl had no effect on blood pressure or any other measured variables at rest (i.e., prior to cycling), further supporting the absence of central cardiovascular effects.

With respect to the use of a >2 SD criterion for ventilatory responses, we agree that this represents a statistical threshold rather than a physiologically-validated benchmark. This approach was used as a pragmatic method to identify atypical ventilatory responses

indicative of potential cephalad migration, consistent with previous work using intrathecal fentanyl.

Taken together, while the experimental data are robust and the ventilatory conclusions are generally supported, the interpretation of the cardiovascular findings raises a fundamental conceptual concern that substantially limits the validity of the conclusions regarding cardiovascular regulation. In its present form, the mechanistic interpretation requires careful reconsideration, and a more explicit and integrative discussion of alternative mechanisms consistent with closed-loop blood pressure regulation would be necessary to adequately support the conclusions.

We appreciate this suggestion and have revised the Discussion to provide a more integrative interpretation of the cardiovascular findings. Specifically, we now:

- Clarify that the reduction in MAP reflects the net haemodynamic consequence of attenuated afferent input, stating that "the reduction in MAP observed with fentanyl is best interpreted as the net haemodynamic consequence of attenuated afferent input, reflecting integrated changes in vascular and central cardiovascular control rather than a direct effect of afferent feedback alone."

- Emphasise a predominant role for changes in peripheral vascular resistance, supported by the DBP-driven reduction in MAP, stating that "the greater reduction in MAP likely reflects an increased reliance on afferent-mediated sympathetic vasoconstrictor support to maintain peripheral vascular resistance when oxygen availability is reduced."

- Address the dissociation between HR and MAP, highlighting that the absence of a compensatory tachycardia suggests limited chronotropic contribution alongside competing influences of central command and reduced sympathetic drive, stating that "Given the fall in MAP, a compensatory baroreflex-mediated tachycardia might be expected; however, HR was only slightly reduced, suggesting that the haemodynamic effects of afferent blockade were not primarily mediated via chronotropic mechanisms. This dissociation between HR and MAP is therefore consistent with a dominant reduction in peripheral vascular resistance, alongside competing influences of reduced sympathetic drive and maintained central command on cardiac control."

- Avoid attributing the response to a single mechanism, instead situating the findings within the context of integrated cardiovascular regulation during exercise.

We believe these revisions provide a more physiologically coherent interpretation while remaining consistent with the experimental data.

Referee #2:

The study by Jenkins and colleagues investigates the contribution of group III/IV skeletal muscle afferents to the cardiovascular, respiratory and metabolic responses to leg cycling

exercise in acute (PIO2=90 mmHg) and chronic (7-9 days at 3,600m, PIO2=90 mmHg) hypoxia. These are complex and demanding experiments undertaken by researchers with a strong track record in undertaken such invasive human studies. The line of enquiry followed builds authentically upon previous work by the team investigating the functional significance of group III/IV skeletal muscle afferents using intrathecal fentanyl (e.g., Amman) and the physiological responses to conditions of low oxygen (e.g., Ainslie and many others). The manuscript is well written, and care has been taken to clearly communicate this rich data set. I have only relatively minor comments:

We thank the referee for their positive evaluation of the manuscript and for their constructive comments.

-The experimental design is very complex involving three exercise intensities (~90W, ~135W, ~185W), two conditions (saline, fentanyl), and three trials (normoxia, sea level hypoxia, high altitude hypoxia). Please can the authors clarify the rationale for including four fixed effects (i.e., intensity, condition, PIO2, acclimatization) in the linear mixed-effects model? Specifically, the practice of comparing normoxia, sea level hypoxia, high altitude hypoxia in terms of both PIO2 and acclimatization fixed-effects. In addition, while it is recognised that conducting such studies on n=14 individuals is a considerable undertaking, it should be considered whether more subtle interactions might have been missed due to a lack of statistical power. Also, why where "independent t-tests" used to examine the effects of fentanyl - as the same individuals are studied in both conditions presumably dependent (paired-samples) t-test would be appropriate.

We thank the referee for this thoughtful comment and the opportunity to clarify our analytical approach.

PIO_2 and acclimatisation were modelled as separate fixed effects to reflect the structure of the experimental design. Specifically, PIO_2 captures the acute effect of reduced inspired oxygen (normoxia vs hypoxia at sea level), whereas acclimatisation captures the additional effect of chronic hypoxic exposure (high-altitude hypoxia vs sea-level hypoxia). This approach allowed us to isolate the effects of acute hypoxia and acclimatisation using a priori planned contrasts, rather than treating the three environments as a single factor.

Intensity was included as a fixed effect to account for systematic differences in physiological responses across exercise workloads. As no higher-order interactions involving intensity were observed, fentanyl–control effects were averaged across intensities to simplify interpretation.

We agree that the sample size may limit the detection of more subtle higher-order interactions. However, our analytical approach was guided by a priori hypotheses and planned contrasts focused on the primary physiological questions, rather than exhaustive interrogation of all interaction terms.

We also thank the referee for noting the description of the resting analyses. Paired (within-subject) t-tests were used to compare fentanyl and control conditions at rest; the term

“independent” was used to indicate that these analyses were conducted separately from the mixed-effects models. This has now been clarified in the revised manuscript.

-Introduction lines 114-125, do not seem necessary. The introduction is already quite long, patients are not being studied, and the argument being made is arguably tangential. Given how well the preceding section is set up this is distracting and it would probably be preferable to omit and lead straight into the aims/hypotheses paragraph.

We thank the referee for this suggestion. We agree that this section was not essential to the central rationale of the study and have removed it to improve the focus and flow of the Introduction.

-Why was a hyperoxia trial performed at high altitude (line 217), but the data are not included? (If included, I could understand better why four fixed-factors were included).

The hyperoxia trial was included at high altitude to preserve consistency in the experimental structure and minimise potential order effects across conditions. However, these data were not included in the present analyses, as exercising in hyperoxia following acclimatisation represents a non-physiological condition that falls outside the primary aims of the study, which focused on responses to normoxia and hypoxia.

Including the hyperoxia condition would also have increased the complexity of the statistical model without directly addressing the central research questions. For these reasons, we elected not to include these data in the current manuscript.

-Results. It is striking how carefully SaO_2 (%) is maintained at rest for sea level and high-altitude trials and yet exercise under both hypoxic conditions yields remarkable individual differences in the responses. Given the authors conclusions (e.g., 303-305), is this inter-individual variability related to variations in the fentanyl-induced suppression of ventilation?

We thank the referee for this observation. Substantial inter-individual variability in arterial oxygen saturation during hypoxic exercise is well established, with previous work demonstrating marked differences in the magnitude of exercise-induced arterial hypoxaemia between individuals (Dempsey & Wagner, 1999; Dominelli et al., 2013). In the present study, a similar degree of variability was evident in the control condition, suggesting this reflects inherent variability in the physiological response to hypoxic exercise rather than a specific effect of fentanyl. While fentanyl consistently reduced ventilation and arterial oxygenation at the group level, we did not observe evidence that it increased the variability of these responses between individuals.

-Figure 7. Please could the authors comment on the differential effects of fentanyl on lactate and $[H^+]$. It is intriguing that while lactate is only increased by fentanyl in acute hypoxia, $[H^+]$ is increased in all three scenarios.

We thank the referee for this observation. The differential effects likely reflect the distinct physiological handling of lactate and hydrogen ions. While lactate accumulation primarily reflects muscle metabolic production and clearance, hydrogen ion concentration is also

influenced by ventilatory control via CO_2 regulation, such that increases in [H^+] can be partially offset by hyperventilation, unlike lactate. As intrathecal fentanyl suppressed ventilation across all conditions, this would be expected to reduce CO_2 clearance and contribute to elevations in [H^+] even in the absence of substantial changes in lactate. We have clarified this point in Section 4.3 of the Discussion.

-Other candidates for including in the "Methodological considerations" section. 1) Three participants were unavailable for sea level testing. 2) Three months separate sea level and high-altitude conditions. 3) Statistical power [as per comment above]. 4) Potential carry over effects of not randomising/counter balancing conditions. 5) Potential contribution of the elevated "pre-exercise" physiological state on the exercise responses reported [SBP is extremely high].

We thank the referee for these suggestions and have addressed each point below.

1) Three participants were unavailable for sea-level testing.

This is correct; three participants did not complete the sea-level phase due to logistical constraints. These individuals were therefore included only in analyses involving high-altitude data. The mixed-effects modelling approach accommodates such missing data without excluding entire participants. As this has already been disclosed in the manuscript, we have not added it to the Methodological considerations.

2) Three months separate sea-level and high-altitude conditions.

We agree this is an important consideration and have now clarified in the Methodological considerations that sea-level and high-altitude trials were separated by ~3 months due to the logistical constraints of the expedition.

3) Statistical power.

We acknowledge that the sample size may limit the detection of more subtle higher-order interactions. However, our analyses were guided by a priori hypotheses and planned contrasts targeting the primary physiological questions, rather than exhaustive interrogation of all interaction terms. We have now clarified this rationale in the manuscript.

4) Potential carry-over effects of not randomising/counterbalancing conditions.

We have now clarified that the fixed order of sea-level followed by high-altitude testing was necessitated by the field-based nature of the study. Importantly, the primary comparisons (fentanyl vs control) were conducted within-environment and were randomised and blinded, which minimises the potential influence of order effects.

5) Elevated pre-exercise physiological state (e.g., SBP).

Pre-exercise values were measured under consistent conditions across trials. The observed values fall within expected ranges for invasive arterial measurements in this context and,

importantly, all primary analyses were based on within-condition comparisons, reducing the influence of baseline differences on the interpretation of exercise responses.

Minor typographical suggestions:

-Given the complexity of the statistical design, it would be helpful if when P values are provided in the text (e.g., abstract, results) the specific interaction referred to is stated. This is often the practice but not applied universally. Also, it would be helpful if the term "condition" were defined more clearly and used more consistently when referring to control/fentanyl, given that there are multiple potential conditions to which the authors could be referring (very minor but will help avoid ambiguity).

We thank the referee for these helpful suggestions. We have now specified the relevant interaction terms alongside reported *p*-values throughout the manuscript (e.g., *condition × PIO_2* or *condition × acclimatisation*) to improve clarity. In addition, we have revised the use of the term "condition" to ensure it is consistently defined as fentanyl vs control and have removed any instances where its meaning could be ambiguous.

-As hypotension technically refers to a specific clinical state, to avoid confusion it would be helpful to reword statements such as "greater hypotensive effect" etc as "greater blood pressure lowering effect" (or similar).

We thank the referee for this suggestion. We have revised the terminology throughout the manuscript to replace "hypotensive" with "blood pressure-lowering" (or equivalent phrasing) to avoid potential ambiguity.

-The phrasing around the rest period was a little ambiguous. In the results section (line 296) "true resting conditions (i.e., prior to the onset of cycling)" are mentioned, which presumably is the "resting baseline" mentioned on line 185, rather than baselines described on lines 197-204. Both baselines are conducted before cycling, and using the term "resting baseline" consistently may avoid ambiguity.

We thank the referee for highlighting this ambiguity. We have now clarified the terminology throughout the manuscript by consistently using "seated rest" to refer to the pre-cycling period, as defined in the Methods, and "resting baseline" to refer to the pre-injection period. This distinction has been applied throughout to improve clarity.

-Please state specific packages used with R for statistical analysis.

We have now specified the R packages used for the statistical analyses in the Methods (Section 2.6).

REFERENCES:

Amann, M., Blain, G. M., Proctor, L. T., Sebranek, J. J., Pegelow, D. F., & Dempsey, J. A. (2010). Group III and IV muscle afferents contribute to ventilatory and

cardiovascular response to rhythmic exercise in humans. *Journal of Applied Physiology*, *109*(4), 966-976.

Dempsey, J. A., & Wagner, P. D. (1999). Exercise-induced arterial hypoxemia. *Journal of Applied Physiology*, *87*(6), 1997-2006. https://doi.org/10.1152/jappl.1999.87.6.1997

Dominelli, P. B., Foster, G. E., Dominelli, G. S., Henderson, W. R., Koehle, M. S., McKenzie, D. C., & Sheel, A. W. (2013). Exercise-induced arterial hypoxaemia and the mechanics of breathing in healthy young women. *The Journal of physiology*, *591*(12), 3017-3034.

Hureau, T. J., Weavil, J. C., Thurston, T. S., Broxterman, R. M., Nelson, A. D., Bledsoe, A. D., Jessop, J. E., Richardson, R. S., Wray, D. W., & Amann, M. (2018). Identifying the role of group III/IV muscle afferents in the carotid baroreflex control of mean arterial pressure and heart rate during exercise. *The Journal of physiology*, *596*(8), 1373-1384.

Dear Dr Jenkins,

Re: JP-RP-2026-290914R1 "The role of group III/IV afferent feedback in the ventilatory, pressor, and metabolic responses to exercise in normoxia and hypoxia" by Elliott J Jenkins, Phuong L Ha, Connor A Howe, Jay M.J.R. Carr, Justin A Monteleone, Andrew JM Douglas, Abigail Nicholson, Andrew R Steele, Brydie Lemkes, Jodie Lauren Koep, L. Madden Brewster, Jennifer Duffy, Mathew Ian Bergens Debenham, Tenasia Monaghan, Declan J H Isaak, Jacob McCormick, Brian H Dalton, Chris J McNeil, Jared Baylis, David B MacLeod, Mike Stembridge, Markus Amann, Philip N Ainslie, and Travis D Gibbons

Thank you for submitting your manuscript to The Journal of Physiology. It has been assessed by a Reviewing Editor and by 2 expert referees and we are pleased to tell you that it is acceptable for publication following satisfactory revision.

The referee reports are copied at the end of this email.

Please address all the points raised and incorporate all requested revisions or explain in your Response to Referees why a change has not been made. We hope you will find the comments helpful and that you will be able to return your revised manuscript within 4 weeks. If you require longer than this, please contact journal staff: jp@physoc.org.

Your revised manuscript should be submitted online using the link in your Author Tasks Link Not Available. This link is accessible via your account as Corresponding Author; it is not available to your co-authors. If this presents a problem, please contact journal staff (jp@physoc.org). Image files from the previous version are retained on the system. Please ensure you replace or remove any files that are being revised.

If you do not wish to submit a revised version of your manuscript, you must inform our journal staff (jp@physoc.org) or reply to this email to request withdrawal. Please note that a manuscript must be formally withdrawn from the peer review process at one journal before it may be submitted to another journal.

TRANSPARENT PEER REVIEW POLICY: To improve the transparency of its peer review process The Journal of Physiology publishes online, as supporting information, the peer review history of all articles accepted for publication. Readers will have access to decision letters, including Editors' comments and referee reports, for each version of the manuscript, as well as any author responses to peer review comments. Referees can decide whether or not they wish to be named on the peer review history document.

ABSTRACT FIGURES: Authors are expected to use The Journal's premium BioRender account to create/redraw their Abstract Figures. Information on how to access this account is here: https://physoc.onlinelibrary.wiley.com/journal/14697793/biorender-access.

This will enable Authors to create and download high-resolution figures. If authors have used the free BioRender service, they can use the instructions provided in the link above to download a high-resolution version suitable for publication.

The link provided should only be used for the purposes of this submission. Authors will be charged for figures created on this account if they are not related to this manuscript submission.

LANGUAGE EDITING AND SUPPORT FOR PUBLICATION: If you would like help with English language editing, or other article preparation support, Wiley Editing Services offers expert help, including English Language Editing, as well as translation, manuscript formatting, and figure formatting at www.wileyauthors.com/eeo/preparation. You can also find resources for Preparing Your Article for general guidance about writing and preparing your manuscript at www.wileyauthors.com/eeo/prepresources.

RIGOUR AND REPRODUCIBILITY: We recommend authors consult The Journal's Rigour and Reproducibility requirements to ensure that their manuscript is suitable for publication: https://physoc.onlinelibrary.wiley.com/pb-assets/hub-assets/physoc/documents/TJP-Rigour-and-Reproducibility-Requirements.pdf

REVISION CHECKLIST:

Check that your Methods section conforms to journal policy: https://jp.msubmit.net/cgi-bin/main.plex?form_type=display_requirements#methods

Check that data presented conforms to the statistics policy: https://jp.msubmit.net/cgi-bin/main.plex?form_type=display_requirements#statistics

Upload a full Response to Referees file. To create your 'Response to Referees' copy all the reports, including any comments from the Senior and Reviewing Editors, into a Microsoft Word, or similar, file and respond to each point, using font or background colour to distinguish comments and responses and upload as the required file type.

Please upload two versions of your manuscript text: one with all relevant changes highlighted and one clean version with no

changes tracked. The manuscript file should include all tables and figure legends, but each figure/graph should be uploaded as separate, high-resolution files. The journal is now integrated with Wiley's Image Checking service. For further details, see: https://www.wiley.com/en-us/network/publishing/research-publishing/trending-stories/upholding-image-integrity-wileys-image-screening-service

You may also upload:

- 'Potential Cover Art' for consideration as the issue's cover image
- Appropriate Supporting Information (Video, audio or data set: see https://jp.msubmit.net/cgi-bin/main.plex?form_type=display_requirements#supp).

We look forward to receiving your revised submission.

If you have any queries, please reply to this email and we will be pleased to advise.

Yours sincerely,

Kim Barrett
Senior Editor
The Journal of Physiology

EDITOR COMMENTS

Reviewing Editor:

Thank you for your thorough responses to the previous round of comments. Most issues are resolved satisfactorily. R1 accepts the robust nature of your observations, upon which your conclusions are based, but argues in a fair manner that limitations of the study provide for alternative or co-existing explanations in part that merit acknowledgement, best placed in a limitations section of the discussion. Notwithstanding the data, the strength of the authors' conviction could be tempered a little by acknowledging the possibility of 'central effects' as argued by R1. I conclude this is a fair request and the peer review report will serve as an additional resource for readers on the evolution of the narrative.

REFEREE COMMENTS

Referee #1:

The authors have made a clear effort to revise the Discussion and adopt a more integrative framing, which is appreciated. However, the central concern remains only partially addressed. The magnitude of the reduction in arterial pressure following group III/IV afferent blockade remains difficult to reconcile with the established integrative model of blood pressure regulation during exercise, and it is unclear why such a large suppression of the pressor response is observed compared to prior human studies.

In addition, the concern regarding potential cephalad migration of fentanyl appears only partly resolved. The inclusion of heart rate responses during the arm-crank control experiment is helpful, but does not directly address arterial pressure regulation. As acknowledged in the Methods, arterial pressure data in this condition were not interpretable, limiting the ability to exclude subtle central cardiovascular effects. The absence of effects at rest also does not preclude central influences during exercise.

These limitations should be explicitly acknowledged, particularly the lack of reliable arterial pressure measurements in the control condition and the indirect nature of the approach used to exclude central effects.

Referee #2:

Thank you for addressing the points raised in my original review.

END OF COMMENTS

Dear Dr Jenkins,

Re: JP-RP-2026-290914R1 "The role of group III/IV afferent feedback in the ventilatory, pressor, and metabolic responses to exercise in normoxia and hypoxia" by Elliott J Jenkins, Phuong L Ha, Connor A Howe, Jay M.J.R. Carr, Justin A Monteleone, Andrew JM Douglas, Abigail Nicholson, Andrew R Steele, Brydie Lemkes, Jodie Lauren Koep, L. Madden Brewster, Jennifer Duffy, Mathew Ian Bergens Debenham, Tenasia Monaghan, Declan J H Isaak, Jacob McCormick, Brian H Dalton, Chris J McNeil, Jared Baylis, David B MacLeod, Mike Stembridge, Markus Amann, Philip N Ainslie, and Travis D Gibbons

Thank you for submitting your manuscript to The Journal of Physiology. It has been assessed by a Reviewing Editor and by 2 expert referees and we are pleased to tell you that it is acceptable for publication following satisfactory revision.

The referee reports are copied at the end of this email.

Please address all the points raised and incorporate all requested revisions or explain in your Response to Referees why a change has not been made. We hope you will find the comments helpful and that you will be able to return your revised manuscript within 4 weeks. If you require longer than this, please contact journal staff: jp@physoc.org.

Your revised manuscript should be submitted online using the link in your Author Tasks https://jp.msubmit.net/cgi-bin/main.plex?el=A2JS1Hwq2B7baa3F4A9ftdbIadOPxAmSylpFV7ffKdDAZ. This link is accessible via your account as Corresponding Author; it is not available to your co-authors. If this presents a problem, please contact journal staff (jp@physoc.org). Image files from the previous version are retained on the system. Please ensure you replace or remove any files that are being revised.

If you do not wish to submit a revised version of your manuscript, you must inform our journal staff (jp@physoc.org) or reply to this email to request withdrawal. Please note that a manuscript must be formally withdrawn from the peer review process at one journal before it may be submitted to another journal.

TRANSPARENT PEER REVIEW POLICY: To improve the transparency of its peer review process The Journal of Physiology publishes online, as supporting information, the peer review history of all articles accepted for publication. Readers will have access to decision letters, including Editors' comments and referee reports, for each version of the manuscript, as well as any author responses to peer review comments. Referees can decide whether or not they wish to be named on the peer review history document.

ABSTRACT FIGURES: Authors are expected to use The Journal's premium BioRender account to create/redraw their Abstract Figures. Information on how to access this account is here: https://physoc.onlinelibrary.wiley.com/journal/14697793/biorender-access.

This will enable Authors to create and download high-resolution figures. If authors have used the free BioRender service, they can use the instructions provided in the link above to

download a high-resolution version suitable for publication.

The link provided should only be used for the purposes of this submission. Authors will be charged for figures created on this account if they are not related to this manuscript submission.

LANGUAGE EDITING AND SUPPORT FOR PUBLICATION: If you would like help with English language editing, or other article preparation support, Wiley Editing Services offers expert help, including English Language Editing, as well as translation, manuscript formatting, and figure formatting at www.wileyauthors.com/eeo/preparation. You can also find resources for Preparing Your Article for general guidance about writing and preparing your manuscript at www.wileyauthors.com/eeo/prepresources.

RIGOUR AND REPRODUCIBILITY: We recommend authors consult The Journal's Rigour and Reproducibility requirements to ensure that their manuscript is suitable for publication: https://physoc.onlinelibrary.wiley.com/pb-assets/hub-assets/physoc/documents/TJP-Rigour-and-Reproducibility-Requirements.pdf

REVISION CHECKLIST:

Check that your Methods section conforms to journal policy: https://jp.msubmit.net/cgi-bin/main.plex?form_type=display_requirements#methods

Check that data presented conforms to the statistics policy: https://jp.msubmit.net/cgi-bin/main.plex?form_type=display_requirements#statistics

Upload a full Response to Referees file. To create your 'Response to Referees' copy all the reports, including any comments from the Senior and Reviewing Editors, into a Microsoft Word, or similar, file and respond to each point, using font or background colour to distinguish comments and responses and upload as the required file type.

Please upload two versions of your manuscript text: one with all relevant changes highlighted and one clean version with no changes tracked. The manuscript file should include all tables and figure legends, but each figure/graph should be uploaded as separate, high-resolution files. The journal is now integrated with Wiley's Image Checking service. For further details, see: https://www.wiley.com/en-us/network/publishing/research-publishing/trending-stories/upholding-image-integrity-wileys-image-screening-service

You may also upload:

- 'Potential Cover Art' for consideration as the issue's cover image
- Appropriate Supporting Information (Video, audio or data set: see https://jp.msubmit.net/cgi-bin/main.plex?form_type=display_requirements#supp).

We look forward to receiving your revised submission.

If you have any queries, please reply to this email and we will be pleased to advise.

Yours sincerely,

Kim Barrett
Senior Editor
The Journal of Physiology

EDITOR COMMENTS

Reviewing Editor:

Thank you for your thorough responses to the previous round of comments. Most issues are resolved satisfactorily. R1 accepts the robust nature of your observations, upon which your conclusions are based, but argues in a fair manner that limitations of the study provide for alternative or co-existing explanations in part that merit acknowledgement, best placed in a limitations section of the discussion. Notwithstanding the data, the strength of the authors' conviction could be tempered a little by acknowledging the possibility of 'central effects' as argued by R1. I conclude this is a fair request and the peer review report will serve as an additional resource for readers on the evolution of the narrative.

We thank the Reviewing Editor for their thoughtful assessment of the revised manuscript and our responses to the previous round of comments. In response to these final comments, we have revised the methodological considerations section to more explicitly acknowledge the limitations associated with excluding potential central effects of intrathecal fentanyl. Specifically, we now recognise the indirect nature of the approach used to assess cephalad drug migration and the absence of interpretable arterial pressure measurements during the arm-crank control condition. We hope these revisions satisfactorily address the remaining concerns.

____

REFEREE COMMENTS

Referee #1:

The authors have made a clear effort to revise the Discussion and adopt a more integrative framing, which is appreciated. However, the central concern remains only partially addressed. The magnitude of the reduction in arterial pressure following group III/IV afferent blockade remains difficult to reconcile with the established integrative model of blood pressure regulation during exercise, and it is unclear why such a large suppression of the pressor response is observed compared to prior human studies.

In addition, the concern regarding potential cephalad migration of fentanyl appears only partly resolved. The inclusion of heart rate responses during the arm-crank control experiment is helpful, but does not directly address arterial pressure regulation. As acknowledged in the Methods, arterial pressure data in this condition were not interpretable, limiting the ability to exclude subtle central cardiovascular effects. The absence of effects at rest also does not preclude central influences during exercise.

These limitations should be explicitly acknowledged, particularly the lack of reliable arterial pressure measurements in the control condition and the indirect nature of the approach used to exclude central effects.

We thank the referee for these thoughtful comments and appreciate the continued emphasis on appropriately contextualising the cardiovascular findings and their mechanistic interpretation.

Accordingly, we have revised the methodological considerations section to more clearly recognise the limitations associated with excluding potential central effects of intrathecal fentanyl. Specifically, we now state:

"Although several observations argue against cephalad migration of fentanyl (including the absence of resting ventilatory or cardiovascular effects, and the lack of suppression of $\dot{V}_E$, $PaCO_2$, $\dot{V}_E/VCO_2$, or HR during the arm-crank control experiment), the approach used to exclude central effects was indirect. Arterial pressure measurements obtained during the arm-crank control condition were not interpretable due to movement artefact, preventing direct assessment of blood pressure regulation in this setting. Consequently, subtle central cardiovascular influences during exercise cannot be definitively excluded."

With respect to the magnitude of the attenuation in exercise MAP following fentanyl administration, we would like to note that this is, to our knowledge, the first study to utilise intrathecal fentanyl during upright cycling exercise in hypoxia and following acclimatisation. As such, direct comparison with previous human studies investigating MAP responses to group III/IV afferent blockade should be made cautiously, particularly given differences in exercise modality, body position, environmental stress, and arterial pressure measurement approaches across studies.

Referee #2:

Thank you for addressing the points raised in my original review.

We thank the referee for their constructive comments and for their careful consideration of the manuscript throughout the review process.

END OF COMMENTS

Dear Mr Jenkins,

Re: JP-RP-2026-290914R2 "The role of group III/IV afferent feedback in the ventilatory, pressor, and metabolic responses to exercise in normoxia and hypoxia" by Elliott J Jenkins, Phuong L Ha, Connor A Howe, Jay M.J.R. Carr, Justin A Monteleone, Andrew JM Douglas, Abigail Nicholson, Andrew R Steele, Brydie Lemkes, Jodie Lauren Koep, L. Madden Brewster, Jennifer Duffy, Mathew Ian Bergens Debenham, Tenasia Monaghan, Declan J H Isaak, Jacob McCormick, Brian H Dalton, Chris J McNeil, Jared Baylis, David B MacLeod, Mike Stembridge, Markus Amann, Philip N Ainslie, and Travis D Gibbons

We are pleased to tell you that your paper has been accepted for publication in The Journal of Physiology.

The last Word (or similar) version of the manuscript provided will be used by the Production Editor to prepare your proof. When this is ready you will receive an email containing a link to Wiley's Online Proofing System. The proof should be thoroughly checked and corrected as promptly as possible.

Authors should note that it is too late at this point to offer corrections prior to proofreading. Major corrections at proof stage, such as changes to figures, will be referred to the Editors for approval before they can be incorporated. Only minor changes, such as to style and consistency, should be made at proof stage. Changes that need to be made after proof stage will usually require a formal correction notice.

All queries at proof stage should be sent to: TJP@wiley.com.

Following copyediting and typesetting the accepted version will be published online.

Yours sincerely,

Kim Barrett
Senior Editor
The Journal of Physiology

IMPORTANT POINTS TO NOTE FOLLOWING ACCEPTANCE OF YOUR PAPER:

• IMPORTANT NOTICE ABOUT OPEN ACCESS: To assist authors whose funding agencies mandate immediate public access to published research findings, The Journal of Physiology allows authors to pay an Open Access (OA) fee to have their papers made freely available immediately on publication.

The Corresponding Author will receive an email from Wiley with details on how to register or log in to Wiley Authors where you will be able to place an order.

• You can check if your funder or institution has a Wiley Open Access Account here:
https://authors.wiley.com/author-resources/Journal-Authors/open-access/author-compliance-tool.html

• TRANSPARENT PEER REVIEW POLICY: To improve the transparency of its peer review process, The Journal of Physiology publishes online as supporting information the peer review history of all articles accepted for publication. Readers will have access to decision letters, including Editors' comments and referee reports, for each version of the manuscript as well as any author responses to peer review comments. Referees can decide whether or not they wish to be named on the peer review history document.

• You can help your research get the attention it deserves! Check out Wiley's free Promotion Guide for best-practice recommendations for promoting your work at: www.wileyauthors.com/eeo/guide. You can learn more about Wiley Editing Services which offers professional video, design, and writing services to create shareable video abstracts, infographics, conference posters, lay summaries, and research news stories for your research at: www.wileyauthors.com/eeo/promotion.

• If you would like to receive our 'Research Roundup', a monthly newsletter highlighting the cutting-edge research published in The Physiological Society's family of journals (The Journal of Physiology, Experimental Physiology, Physiological Reports, The Journal of Nutritional Physiology and The Journal of Precision Medicine: Health and Disease), please click this link, fill in your name and email address and select 'Research Roundup':
https://www.physoc.org/journals-and-media/membernews

EDITOR COMMENTS

Reviewing Editor:

Thank you for addressing these final points and for making final revisions to the text. All points have now been addressed and I thank you for your positive engagement with the peer review process. Congratulations on an excellent study!
